# Arthropod Food Webs in the Foreland of a Retreating Greenland Glacier: Integrating Molecular Gut Content Analysis With Structural Equation Modelling

**DOI:** 10.1002/ece3.70687

**Published:** 2024-12-17

**Authors:** Ejgil Gravesen, Lenka Dušátková, Kacie J. Athey, Jiayi Qin, Paul Henning Krogh

**Affiliations:** ^1^ Independent Researcher Åbyhøj Denmark; ^2^ Department of Botany and Zoology, Faculty of Science Masaryk University Brno Czech Republic; ^3^ Crop Sciences University of Illinois at Urbana‐Champaign Urbana Illinois USA; ^4^ Novo Nordisk København Ø Denmark; ^5^ Department of Ecoscience Aarhus University Aarhus C Denmark

**Keywords:** *Aclastus borealis*, antipredatory behavior, deglaciation, detritivores, extra‐guild prey, *Isotoma anglicana*, *Mitopus morio*, NDVI, *Nebria rufescens*, pioneer vegetation

## Abstract

The Arctic has warmed nearly four times faster than the global average since 1979, resulting in rapid glacier retreat and exposing new glacier forelands. These forelands offer unique experimental settings to explore how global warming impacts ecosystems, particularly for highly climate‐sensitive arthropods. Understanding these impacts can help anticipate future biodiversity and ecosystem changes under ongoing warming scenarios. In this study, we integrate data on arthropod diversity from DNA gut content analysis—offering insight into predator diets—with quantitative measures of arthropod activity‐density at a Greenland glacier foreland using Structural Equation Modelling (SEM). Our SEM analysis reveals both bottom‐up and top‐down controlled food chains. Bottom‐up control, linked to sit‐and‐wait predator behavior, was prominent for spider and harvestman populations, while top‐down control, associated with active search behavior, was key for ground beetle populations. Bottom‐up controlled dynamics predominated during the early stages of vegetation succession, while top‐down mechanisms dominated in later successional stages further from the glacier, driven largely by increasing temperatures. In advanced successional stages, top‐down cascades intensify intraguild predation (IGP) among arthropod predators. This is especially evident in the linyphiid spider *Collinsia holmgreni*, whose diet included other linyphiid and lycosid spiders, reflecting high IGP. The IGP ratio in *C. holmgreni* negatively correlated with the activity‐density of ground‐dwelling prey, likely contributing to the local decline and possible extinction of this cold‐adapted species in warmer, late‐succession habitats where lycosid spiders dominate. These findings suggest that sustained warming and associated shifts in food web dynamics could lead to the loss of cold‐adapted species, while brief warm events may temporarily impact populations without lasting extinction effects.

## Graphical abstract

This study investigates the ecological dynamics of arthropod predators across a transect of an Arctic glacier foreland, to understand how climate change impacts predator–prey interactions. By integrating ecological observations with DNA metabarcoding, our research elucidates the role of the key prey species in shaping spider populations in these habitats. The findings highlight the importance of interdisciplinary approaches in unraveling complex ecological processes and inform conservation strategies in the face of ongoing environmental changes.

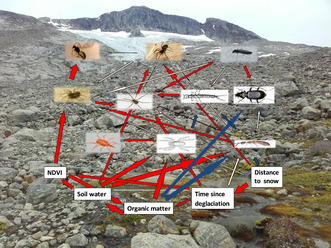

## 1 Introduction

The Arctic and Antarctic regions are impacted by climate change, experiencing some of the fastest temperature changes observed on Earth (Turner et al. 2014; Overland et al. 2019). The Arctic has warmed nearly four times faster than the global average since 1979 (Rantanen et al. 2022), leading to major impacts on terrestrial ecosystems—particularly arthropods, which are highly sensitive to climate change (Høye 2020). Moreover, Voigt et al. (2003) found that sensitivity to climate variations, such as temperature and precipitation changes, increased significantly with the trophic level of arthropods. In the Arctic, arthropods are the dominant terrestrial animal group, with insects representing 60%–80% of the described terrestrial animal diversity (Loboda et al. 2018). Bowden et al. (2018) and Loboda et al. (2018) showed declining abundances of linyphiid spiders and muscid flies in the Arctic in response to rising temperatures. A direct consequence of global warming is retreating glaciers exposing glacier forelands in front of the glacier snouts. These glacier forelands are perfect experimental areas in relation to the consequences of global warming for arthropods as it is possible to observe the population dynamics and food webs in relation to warming within a limited area. In these dynamic environments, cold‐adapted species like linyphiid spiders are particularly well suited to the cold, wet conditions near glacier forelands. Linyphiid spiders typically employ a sit‐and‐wait strategy, relying on the abundance of available prey and remaining stationary until prey comes within reach (Nentwig et al. 2022). In contrast, lycosid spiders, which dominate the warmer habitats of the Kobbefjord Valley, are better adapted to sunnier and more stable environments. They utilize active hunting strategies, where they actively search for and pursue prey, allowing them to exert greater control over prey populations in their environment (Nentwig et al. 2022). These contrasting adaptations allow for a clear comparison of how different arthropod groups respond to shifting climates covering years with distinct winter and spring temperature profiles. The results of such research may give important information about what may happen in the future in relation to global warming scenarios. Climate change is causing glaciers to retreat in Greenland and elsewhere, resulting in a rapid colonization of populations of surface living arthropods. Increase of organic matter as a result of plant colonization and the increase of plant production away from the glacier snout is expected to increase the abundance of Collembola and linyphiid spiders (Patrick, Kershner, and Fraser 2012; El‐Nabawy et al. 2016). This development in the early stage of succession (Ficetola et al. 2021), can be explained by bottom‐up mechanisms found in a Norwegian alpine region (Hågvar and Klanderud 2009) with predators profiting from these first colonizers (Sint et al. 2019).

The colonization of tiny pioneer plants and bacteria (Schütte et al. 2009; Ciccazzo et al. 2016) and the concomitant development of a litter layer close to the glacier snout is supposed to be the food resource for the pioneer colonizing detritivores (Hågvar and Klanderud 2009; Koltz, Classen, and Wright 2018), such as the collembolan, *Isotoma anglicana* Lubbock 1862, which has physiological adaptations to cold conditions (Sørensen and Holmstrup 2011).

Predation by arthropods has been shown to be affected by low temperature (Kruse, Toft, and Sunderland 2008). For example, predatory mites exposed to cold reduce their predation rates and acclimatization to low temperatures improves starvation tolerance (Jensen et al. 2017, 2018, 2019). Higher temperatures resulting from climate change may intensify top‐down effects by elevating the metabolic rates of predators, which in turn enhances processes such as foraging activity (Hoekman 2010). Therefore, we anticipate greater control of prey populations by arthropod predators further away from the glacier snout. Consequently, if predation rates are higher in areas further from the glacier than in those closer to the glacier snout, this will lead to a decline in prey populations (Sohlström et al. 2022). This development results in top‐down control during the later stages of succession, as active arthropod predators may significantly reduce prey populations (Gratton and Denno 2003).

Increasing predation away from the glacier snout—leading to low prey availability—will increase the strength of the top‐down effect in this low production Arctic ecosystem and lead to increased intraguild predation (IGP) because of starvation (König, Kaufmann, and Scheu 2011; Lucas and Rosenheim 2011). Indeed, Raso et al. (2014) found high IGP in glacier forelands among all arthropod predators in the Alps. Cannibalism and IGP has the potential to maintain arthropod predator populations during periods of low abundance of prey at lower trophic levels, thereby promoting trophic cascades in the future when conditions change (Wise 2006).

Rall et al. (2010) found that warming could either strongly stabilize or destabilize arthropod populations and food webs by changing the interaction strengths between predators and their prey. Rall et al. (2010) argue that warming of natural ecosystems could increase population stability with a higher probability of returning to the same equilibrium density after a small perturbation—for example, extreme weather events—as such short‐lasting weather events may temporarily reduce the predator and prey abundances but not lead to extinction of any of these groups. In relation to a longer time scale, warming may also lead to decreasing ingestion efficiencies which may lead to starvation and which will further lead to higher IGP ratios (Lucas and Rosenheim 2011)—increasing the risk of extinction among the predators.

This study examines arthropod species composition across a climatic gradient in the glacier foreland region, comparing sites with different vegetation and temperature conditions. Notably, we observed variations in winter and spring temperatures across the years, with 2015 and 2017 being relatively cold and 2016 experiencing warmer winter and spring conditions.

We will also look at the arthropod population dynamics change away from the glacier snout when the temperatures increase and go further into details regarding the relationships between specific predators and their potential prey.

To explore specific relationships between predators and their prey, we have used a combination of Structural Equation Modeling (SEM) with molecular gut content analysis to reveal the structuring forces, for example, bottom‐up and top‐down mechanisms, within the explored arthropod food webs (Shao et al. 2015) The SEM models are based on the arthropod activity‐densities, the prey composition in the guts of the predators as well as important environmental variables characterizing the glacier foreland. Furthermore, the SEM modeling is supported by the existing literature.

Bottom‐up mechanisms are indicated by concurrent increases in both predator and prey populations as temperatures rise, suggesting that more resources (e.g., organic matter) support both groups. In contrast, top‐down mechanisms are characterized by increasing predator populations accompanied by declining prey populations, indicating that predators exert more control over prey numbers as temperatures increase. Positive and negative correlations found in SEM are indications of bottom‐up and top‐down food chains between a specific predator and a potential prey where the energy may be transferred from a prey to a predator.

By employing SEM modeling, we explore stabilization and destabilization mechanisms in relation to linyphiid population dynamics, with a focus on IGP and potential antipredatory behaviors among linyphiid spiders. To illustrate these mechanisms, we use examples from other well‐studied ecosystems—for example, temperate agricultural fields. By investigating how arthropod populations develop with increasing distance from the glacier snout and examining the potential influences of both general environmental trends and species‐specific traits (such as hunting strategies and movement patterns), our research aims to deepen the understanding of arthropod succession in the face of climate change. This approach is designed to illuminate the complex interplay between bottom‐up and top‐down processes shaping these ecosystems, leveraging SEM and molecular gut content analysis to explore the structuring forces within arthropod food webs. This lead to the following four main hypotheses:

Hypothesis 1 Cold‐adapted arthropod predators will dominate in the glacier foreland's colder environments, while warm‐adapted species will be more prevalent in the warmer Kobbefjord Valley.

Hypothesis 2 Bottom‐up mechanisms will dominate near the glacier snout, with a shift to top‐down control and increased predation as temperatures rise in late succession habitats further downhill.

Hypothesis 3 Predators' trophic links will vary based on their hunting strategies, with sit‐and‐wait predators depending on prey availability and active hunters controlling prey populations.

Hypothesis 4 Climatic warming will destabilize cold‐adapted predator populations, leading to increased intraguild predation and a risk of extinction for cold‐tolerant species.

## 2 Materials and Methods

### 2.1 Study Area

**FIGURE 1:**
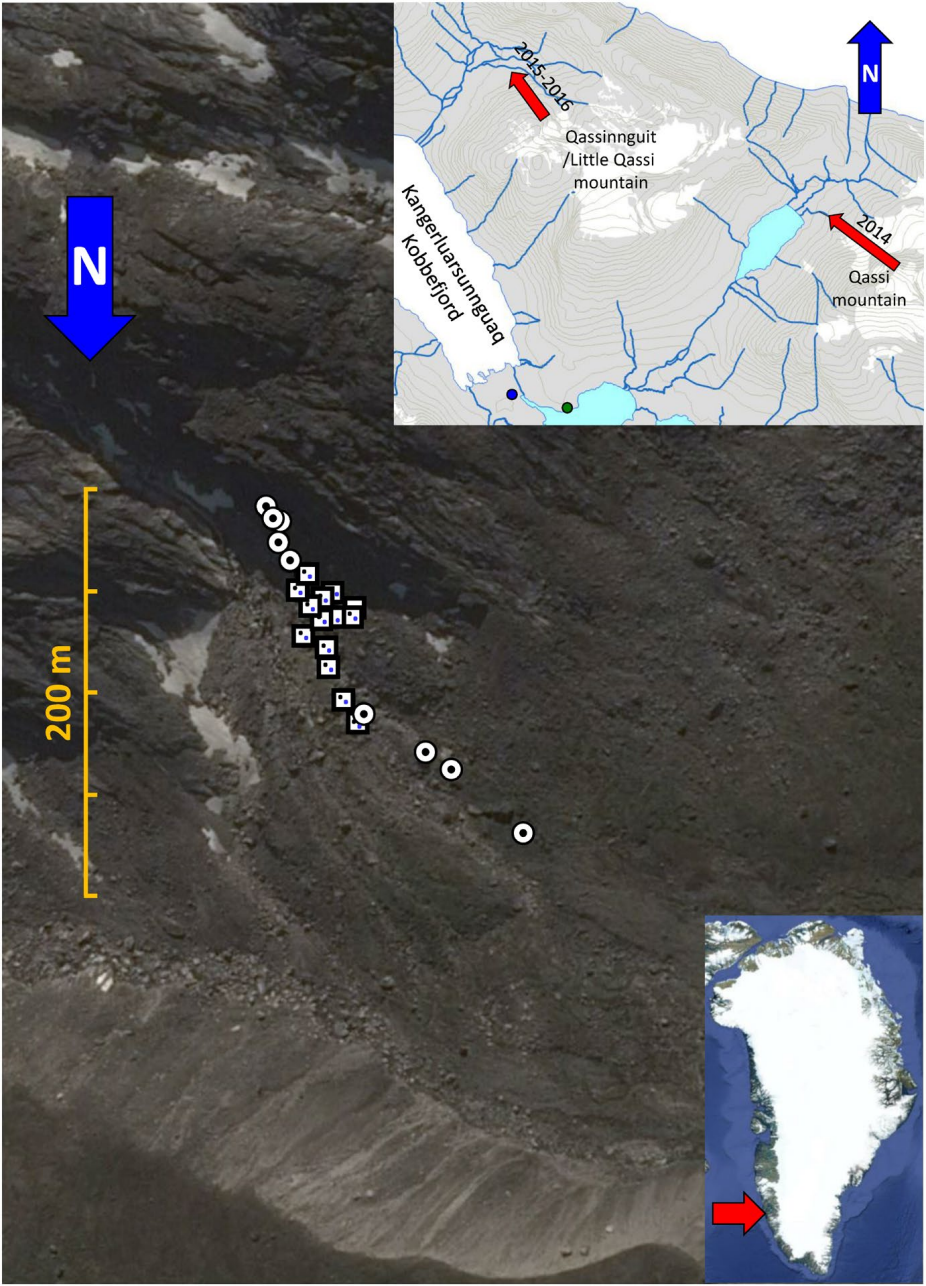
Position of the 23 observation patches along the study transect down the mountain slope of the Little Qassi mountain (Qassinnguit). Upper transect patches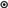, 277–291 MASL, in the shadow of the mountain with one wet pitfall trap at each patch; central transect patches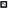, 242–277 MASL, with partly shadow during a midsummer day with a wet pitfall (blue dot) and a dry pitfall trap (black dot) at each patch; lower transect patches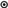, 206–242 MASL, without shadow during a midsummer day, with one wet pitfall trap at each patch. Right hand side red arrows show the position of the whole Kobbefjord area (lower right) and the position of the 2014 study transect within the Kobbefjord area east of the Little Qassi Mountain as well as the crowberry 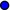 and gray willow 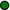 plots in the Kobbefjord valley.

**FIGURE A1:**
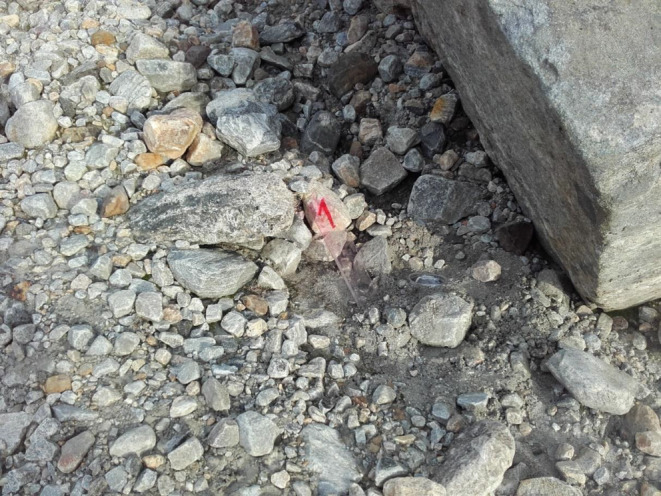
This photo shows the uppermost patch on the Qassinnguit transect without any visible vegetation cover as this area was covered with snow the summer before and there was only little sunlight as there was almost permanent shadow from the mountain to the south. A wet pitfall trap is (barely) visible next to the number “1”. No lichens were found on the rocks at this site.

**FIGURE A2:**
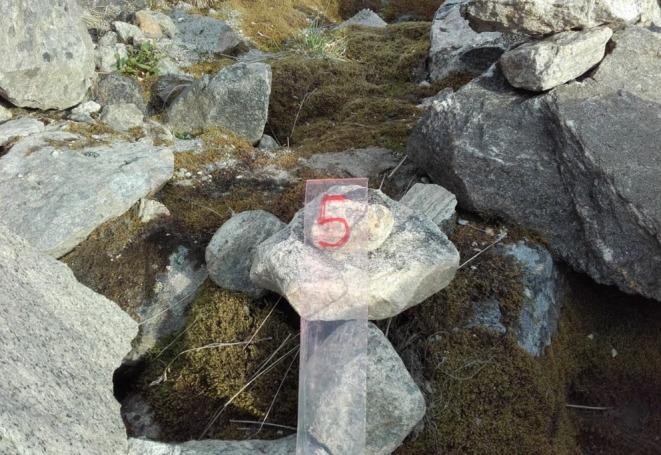
A patch at the upper Qassinnguit transect dominated by mosses.

**FIGURE A3:**
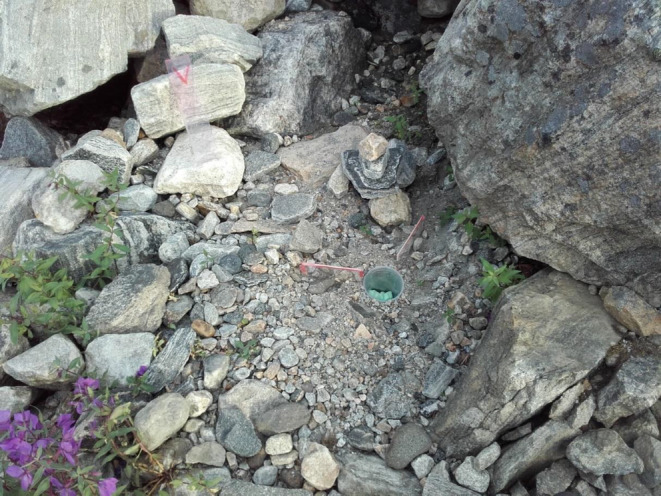
A typical bare soil patch in the central area with no or almost no (pioneer) vegetation cover as the patch may be flooded during snow melt in spring time. In the middle of the photo is a dry pitfall trap half‐filled with green styrofoam chips and X‐shaped guidance barriers.

**FIGURE A4:**
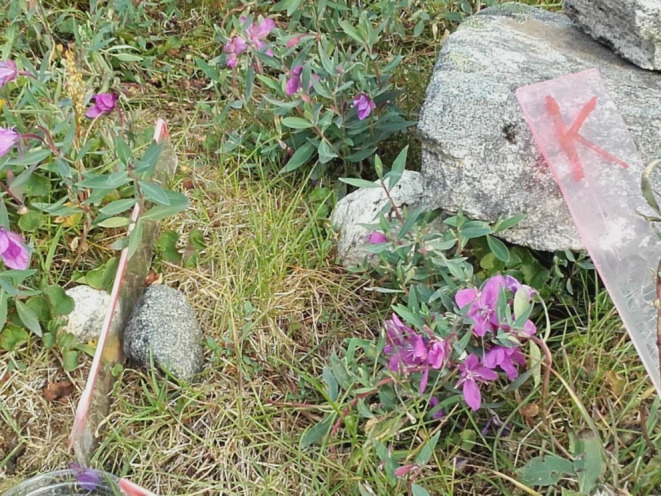
A patch with pioneer vegetation cover in the central area which may have some protection from flooding by melting water during spring time.

**FIGURE A5:**
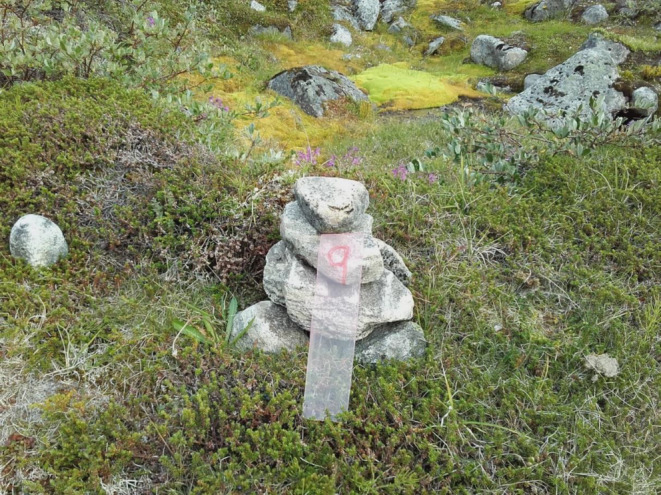
The lowest patch on the Qassinnguit transect with a climax vegetation cover consisting of crowberry in the foreground and grey willow in the background to the left and mosses to the right.

**FIGURE A6:**
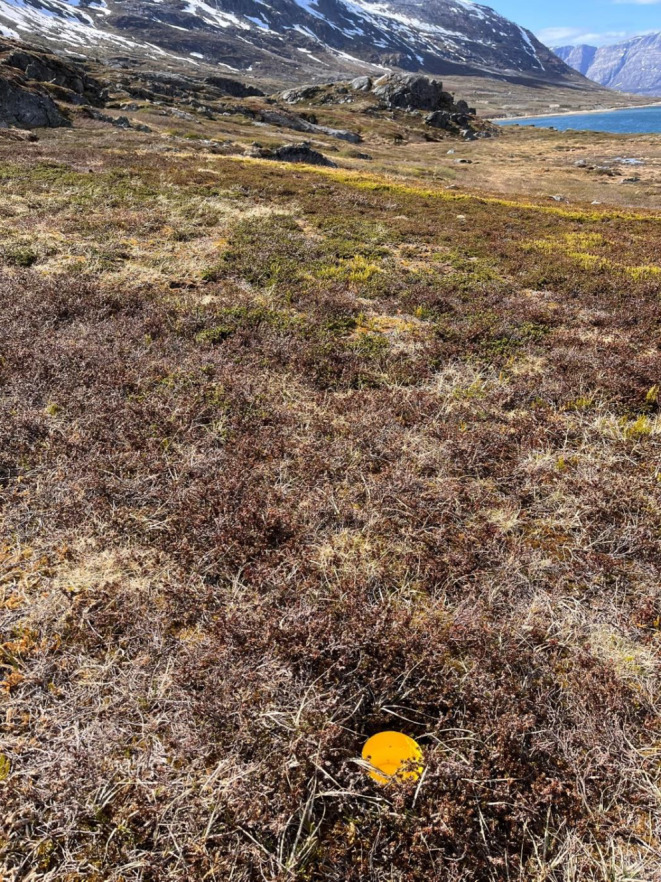
The crowberry climax vegetation habitat in the Kobbefjord valley – with the fjord to the west. This site is exposed to the wind and the sun all day during the summer. A yellow pitfall trap is seen between the low crowberry vegetation layer.

**FIGURE A7:**
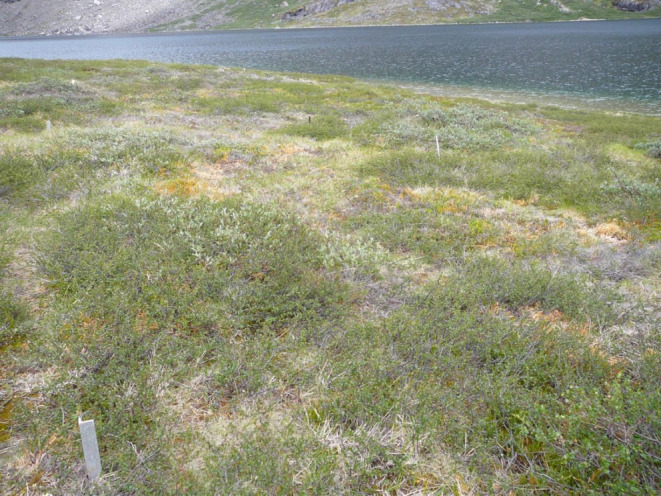
The grey willow climax vegetation habitat in the Kobbefjord valley ‐ with a lake to the south ‐ which is exposed to sunlight all day during the summer.

The main study area was located on the north‐facing slope of the Qassinnguit mountain, approximately 2 km north of Kobbefjord Valley in South‐West Greenland. The transect, studied during the summers of 2015 and 2016, ran downslope the Qassinnguit mountain toward the northwest, across the glacier foreland. During the summer of 2014, a study was conducted on a similar glacier foreland located on the northwestern slope of the Qassi mountain. This site was situated approximately 5 km northeast of Kobbefjord Valley and 2 km southeast of the Qassinnguit glacier foreland. Both the Qassinnguit and Qassi glacier forelands had pioneer vegetation. In the Kobbefjord Valley, two climax vegetation sites (dominated by crowberry and gray willow) were used for comparison during the summers of 2015–2017 (Figure 1). For more details about the geographical locations and locality photos, see Figure A1, A2, A3, A4, A5, A6, A7 and the methods section of the Appendix AM1.

In the central foreland area at the Qassinnguit glacier (Figure 1), all snow had melted by July 1, 2015. However, areas below and above the central transect still experienced snow cover throughout July and into the beginning of August. During the summer of 2016, there was no snow cover in the entire Qassinnguit glacier foreland area—neither on a transect below nor above the central transect.

The Qassinnguit and Qassi glacier forelands experienced longer periods of snow cover compared to the Kobbefjord Valley, due to their higher elevation and reduced exposure to wind and sunlight. In contrast, the climax vegetation sites in the Kobbefjord Valley, situated in open, wind‐exposed areas with all‐day sunshine during summer, saw shorter snow cover durations. The crowberry site, characterized by a relatively low vegetation layer (Figure A6), was the most exposed to wind and sunlight, whereas the gray willow site (Figure A7), featuring tall shrubs that could act as windbreaks, retained its snow cover for longer periods during the spring thaw (Legault and Weiss 2013) For more details about climatic differences between years, see the climatic Table A1.

### 2.2 Environmental Characterization

**FIGURE A8:**
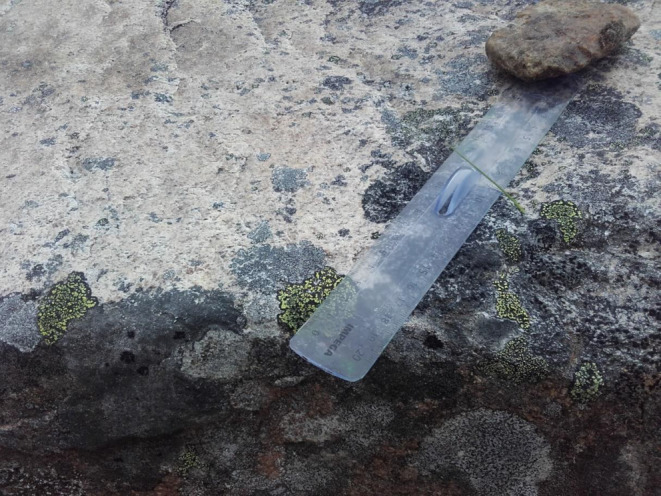
The lichenometric dating method measuring *Rhizocarpon thalli* diameters.

**FIGURE A9:**
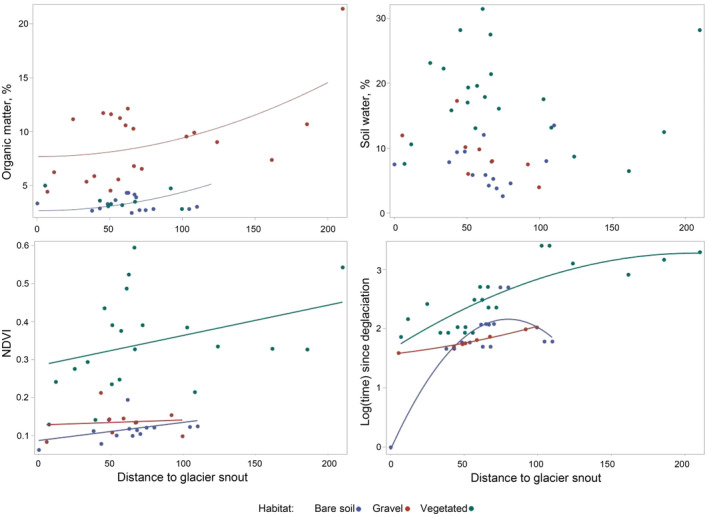
Modelling environmental variables against the distance to glacier snout. The relationship between the distance to the glacier snout down the transect and the four significant environmental variables. The curves are the 2nd degree polynomial fit (*F* < 5%). Missing NDVI values for 2015 were predicted from soil water, organic matter and time since deglaciation.

Soil water and organic matter content was measured for each patch in the glacier foreland in both the summer of 2015 and 2016 and NDVI (Normalized Difference Vegetation Index) measurements (Pettorelli et al. 2011) were taken at each patch in the summer of 2016 (see AM2, Figure A9). NDVI data were obtained for the Kobbefjord climax vegetation habitats from the GEM Database for 2016 (Greenland Ecosystem Monitoring 2020b). NDVI was used to show the arthropod population developments away from the glacier snout at the Qassinnguit glacier foreland (Figure 3) while the distances to the glacier snouts were used in relation to the population development of *I. anglicana* and its potential predators at the Qassinnguit and Qassi glacier forelands (Figure 4). We estimated the time since deglaciation using *Rhizocarpon* thalli growth (Forman et al. 2007) (Table A2, Figure A8, AM2).

### 2.3 Arthropod Sampling

In the summer of 2015, a central transect of the Qassinnguit foreland was surveyed. This was done using a combination of one wet and one dry pitfall trap within each of 21 patches, separated randomly in a group of seven vegetated patches, a group of seven bare soil patches, and a group of seven gravel covered patches (Figure 1, Table A11). The dry pitfall traps were used to collect live arthropod predators for molecular gut content analysis while the wet pitfall traps gave us activity‐density abundances used for quantitative data analysis.

During the summer of 2016, only patches with vegetation and bare soil cover were surveyed with 14 pitfall traps using the same setup as in 2015 except that the gravel covered patches were excluded. A transect was extended above and below the central transect with wet pitfall traps. The upper and lower parts of the transect had five patches and four patches each, respectively, each with one wet pitfall trap (Figure 1). The upper transect patches had bare soil cover with just a little or no sunlight as they were in the shadow of the sun—even at midsummer (Figure A1). Two of the upper transect patches were covered by a mostly permanent snow layer before the summer of 2016 and were situated just next to the glacier snout consisting of permanent snow or ice, hereafter referred to as the glacier snout. The lowest transect patch had a climax vegetation cover, mainly with crowberry, which is a common heather plant (family Ericaceae) in Greenland (Figure A5).

During the summer of 2014, wet pitfall traps were used to survey a 259‐m long transect at a glacier foreland on a northwestern slope of the Qassi mountain (Figure 1). The two climax vegetation habitats, crowberry and willow, each with four replicates in the Kobbefjord Valley were surveyed during the same summer periods as at the glacier forelands of the Qassinnguit and the Qassi mountains from the summer of 2015 till the summer of 2017 (Greenland Ecosystem Monitoring 2020a). For more information about the arthropod sampling, see AM3.

Pitfall trap catches (using wet pitfall traps) measure the activity‐density, reflecting the likelihood of encounters between predators and potential prey animals (Høye and Forchhammer 2008b; Brown and Matthews 2016). We use the term “activity‐density” to describe this measure as it quantifies the movement and density of arthropods in the environment in contrast to area‐based density measures. This data is particularly useful when discussing gut content analysis combined with food preference analysis (Vaughan et al. 2018), and in the context of our two SEM models (SEM1 and SEM2) based on pitfall trapping data from the Qassinnguit glacier foreland. These analyses help in understanding predator–prey dynamics and the overall ecological interactions.

In contrast, DNA gut content analysis provides a qualitative measure by identifying prey found in the guts of predators. Identifying prey in this way is crucial for establishing trophic links between predators and potential prey. The choice of these trophic links, that is, SEM pathways, is further supported by scientific literature and expert judgment.

**FIGURE A10:**
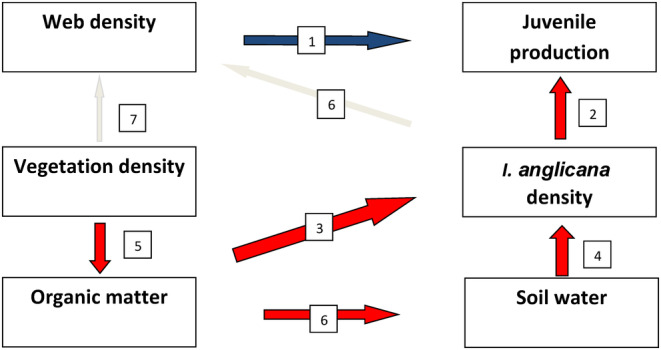
SEM model, SEM3, of wheat‐clover bi‐cropping experiment. The path diagram presents a model for the relationships between linyphiid web density, vegetation density, *I. anglicana* density, linypiid juvenile production, organic matter and soil water content in the top‐soil using Structural Equation Modeling (SEM). Model fit: Chi‐square = 17.65; Probability level = 0.014; df = 7; Sample size = 120. The thickness of each arrow represents the standardized regression weights. Red arrows shows significant positive correlations and blue arrows shows significant negative correlations. Grey arrows shows non‐significant correlations. The numbers at each arrow refers to more detailed information in relation to each correlation in Table A11.

For instance, in the SEM3 diagram illustrating an arthropod food web in Danish experimental wheat fields (Table A10, Figure A10; Gravesen 2008), arthropod abundances are area‐based density estimations only. These three SEM models show how it has been possible to develop SEM within arthropod ecology research, thanks to the development of DNA gut content analysis (Macías‐Hernández et al. 2018).

#### 2.3.1 DNA Gut Content Analysis of Arthropod Predators and Bioinformatics

The DNA extraction and analysis from predators and their potential prey collected in 2015 and 2016 focused on gut content using metabarcoding techniques (AM4) and single‐plex PCR (AM5). The analysis included the application of the MiteMinBar minibarcode (de Groot, Laros, and Geisen 2016) to identify various prey species accurately. To improve the reference COI database used to assign species names to the amplicons during the bioinformatics step, we barcoded a selection of the captured arthropods (Table A4). This molecular approach enhances our understanding of the complex food web dynamics within the Qassinnguit glacier foreland and surrounding areas. Details are included in AM4, AM5.1, and AM5.2.

### 2.4 Data Analysis

A generalized linear mixed model (GLMM) was employed to explore population abundances along a downhill transect in the glacier foreland, examining both bottom‐up and top‐down control mechanisms within the arthropod predator–prey relationships. The analysis included comparisons of arthropod taxa across different years and habitats, specifically pioneer, crowberry, and gray willow habitats, utilizing a GLMM with a Poisson distribution. The Tukey–Kramer test facilitated multiple means comparisons as part of the SAS procedure GLIMMIX (SAS Institute Inc. 2018). Additionally, a Poisson regression was applied to analyze the influence of NDVI and proximity to the glacier snout on selected arthropod taxa, incorporating both linear and second‐degree polynomial models.

The taxonomic richness and Shannon diversity index for all species, predators, and prey alike, were assessed using a linear model that included variables such as position on the transect at three levels (upper, central, and lower), habitat type, and year, along with their interactions, processed through the SAS PROC MIXED procedure (SAS Institute Inc. 2018).

In terms of specific predatory behavior, the paired samples *t*‐test was conducted to compare *I. anglicana* activity‐density between the two summer periods. To analyze predation frequencies, the *econullnetr* package in R was utilized, comparing the observed presence of prey DNA in predator guts against the activity‐density data of prey taxa from pitfall traps, using 95% CIs derived from a null model (Vaughan et al. 2018).

IGP‐ratios for key predators like *Collinsia holmgreni* Thorell 1871, the harvestman, *Mitopus morio* (Fabricius, 1779), and both adult and larval stages of the ground beetle were quantified by determining the percentage of other predator species found in their guts relative to the total number of species present.

Two separate SEM models were developed to further explore these trophic relationships. These models were constructed using SPSS Amos software (Arbuckle 2022), which incorporated hypothesized pathways reflecting trophic interactions from the literature and findings from the DNA gut content analysis. The SEM models highlighted significant relationships and potential influences among the variables, indicating whether positive or negative correlations suggest bottom‐up or top‐down food web dynamics.

Molecular techniques were vital in delineating these food web interactions as traditional visual inspection fails due to many predators being liquid feeders or employing extra oral digestion. These techniques ensure accurate identification of prey in predator guts (Cohen 1995; Eitzinger and Traugott 2011; Whitney et al. 2018). Pairing DNA gut content analysis with activity‐density sampling provided a comprehensive approach to describing the food webs, as predator consumption often does not directly correlate with prey population densities (Athey et al. 2016). A trophic link between a predator and a potential prey was chosen in the two SEM models if the prey was identified in the predator's gut and there was scientific literature support for choosing such a link. Erythraeoidea was not identified in any predator guts and therefore this group of prey was excluded from the SEM model. If there was no gut content analysis done on a predator—for example, Bdelloidea—or a group of predators—for example, “other Araneae”—a trophic link was chosen solely based on the literature or on expert communication. If we found no significant correlation between a predator and a prey—for example, the dipteran family Simuliidae—this group of prey was excluded from the SEM model.

Data were prepared for SEM analysis with transformations appropriate to the statistical models used, selecting the best models based on Chi‐square goodness‐of‐fit statistics, *p*‐values, CMIN/df close to one, Comparative Fit Index (CFI), Root Mean Square Error of Approximation (RMSEA), and Root Mean Square Residual (RMR) (Arbuckle 2022). The overall arthropod food web for the glacier foreland was modeled using wet pitfall activity‐density data pooled from the summers of 2015 and 2016 (SEM1), while the detailed model in relation to the IGP of *C. holmgreni* was based on data sampled during the summer of 2016 (SEM2). These two SEM models are based on DNA gut content analysis performed on arthropod predators sampled with dry pitfall traps during the summer of 2016.

In each SEM analyses, the variances of the error terms associated with the observed variables were fixed to unity. This approach was taken to ensure model identification, as these error terms include unspecified latent variables. While these fixed variances are essential for the proper specification of the models, they are not displayed in the SEM diagrams to maintain clarity. This method allows for meaningful estimation of other model parameters (Arbuckle 2022).

## 3 Results

### 3.1 Activity‐Density of Arthropods in the Qassinnguit Glacier Foreland and in the Climax Vegetation Habitats in the Kobbefjord Valley—Hypothesis 1

Overall, sampling with both wet and dry pitfall traps in the Qassinnguit glacier foreland indicated low predator diversity in 2015 and 2016. Specifically, there was one species of carabid beetle, one species of harvestman, 20 species of spiders, and five suborders/families of Hymenoptera. Among the potential prey, there were 19 families of Diptera, six families of Acari (mites), three families of Collembola, one family of Lepidoptera (Tortricidae), and one species of Aphididae. Table A8 presents the mean activity‐density using wet pitfall trap catch numbers of arthropods collected from both vegetated and bare soil patches during the summers of 2015 and 2016, as well as in the upper and lower transects during the summer of 2016. Notably, six linyphiid spider species were absent, while *C. holmgreni* was rare in the lower transect of the glacier foreland and none of those species were found in the warmer habitats of Kobbefjord (Pétillon, Courtial, and Vernon 2014) (Table A9). Table A5 demonstrates that both predator and prey diversity was highest in the vegetated patches during the summer of 2015, while diversity was particularly low in the bare soil patches near the glacier.

**FIGURE 2:**
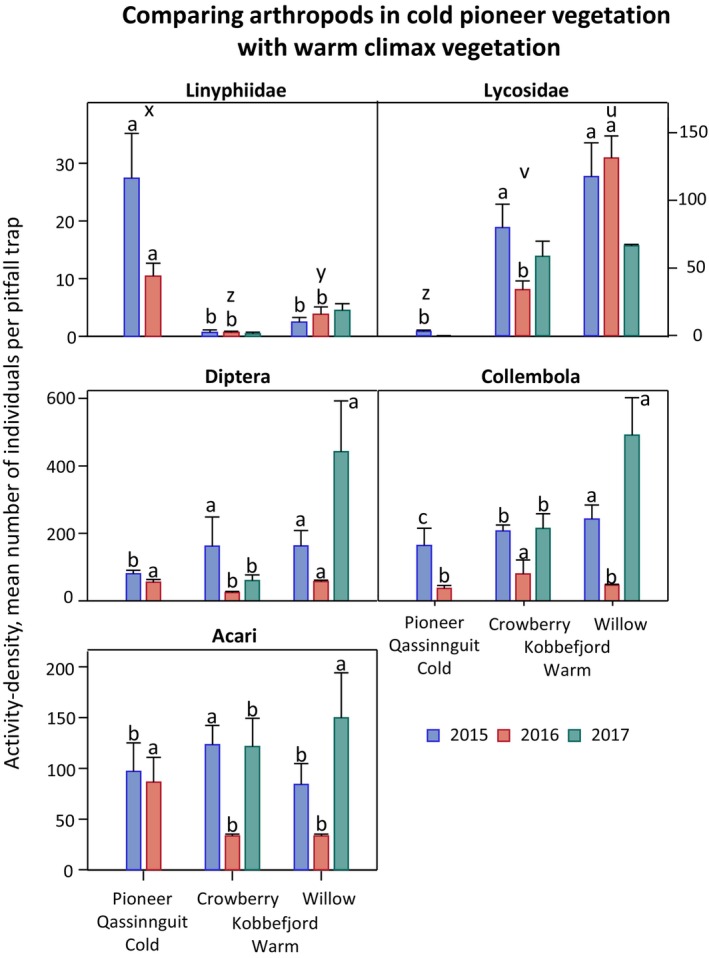
Activity‐densities of Linyphiidae, Lycosidae, and the prey groups—Diptera, Collembola, and Acari—in the Qassinnguit glacier foreland as well as in the warmer climax vegetation habitats (crowberry and gray willow) in the Kobbefjord Valley (Greenland Ecosystem Monitoring 2020a). Different letters (a, b, and c) indicate significant differences between years within habitats, while u, v, x, y, and z indicate significant differences between activity‐densities of linyphiids and lycosids in the three different habitats (pioneer, crowberry, and gray willow) across years.

Eighty‐seven percent and 76% of the spiders sampled in the central transect (in vegetated and bare soil patches) of the glacier foreland during the summer of 2015 and 2016, respectively, were linyphiids while 13% and 0.5% were lycosids in the summer of 2015 and the summer of 2016, respectively (Figure 2). In contrast, the two climax vegetation habitats south of Qassinnguit mountain had the opposite trend. Lycosids accounted for 76%–73% (2015–2016) in crowberry dominated habitat and 74%–85% (2015–2016) in gray willow‐dominated habitat. Linyphiids caught in these habitats were less than 2% of the spider populations (Greenland Ecosystem Monitoring 2020a). Both these climax vegetation types are supposed to have a warmer microclimate than the glacier foreland area on the northern slope of the Qassinnguit mountain as both habitats are exposed to sunlight all day during the summertime.

The mite, collembolan, and dipteran populations were fluctuating at the glacier foreland between the summer of 2015 and the summer of 2016 with significantly lower activity‐density in the summer of 2016. A similar pattern with fluctuating prey populations was seen in the warmer climax vegetation habitats in the Kobbefjord Valley, dominated by crowberry or gray willow while the spider populations were relatively stable between the years as there only was significantly lower mean activity‐densities in 2016 compared to 2015 for the lycosids in the crowberry vegetation habitat in the Kobbefjord Valley while both the linyphiid (in both climax vegetation habitats) and the lycosid populations (in the gray willow habitat) were relatively stable between the different years. Table A9 shows linyphiid species caught in (wet) pitfall traps in the climax vegetation habitats in the Kobbefjord Valley during the summer of 2015 and 2016.

### 3.2 Arthropod Population Developments Away From Glacier—Hypothesis 2

**FIGURE 3:**
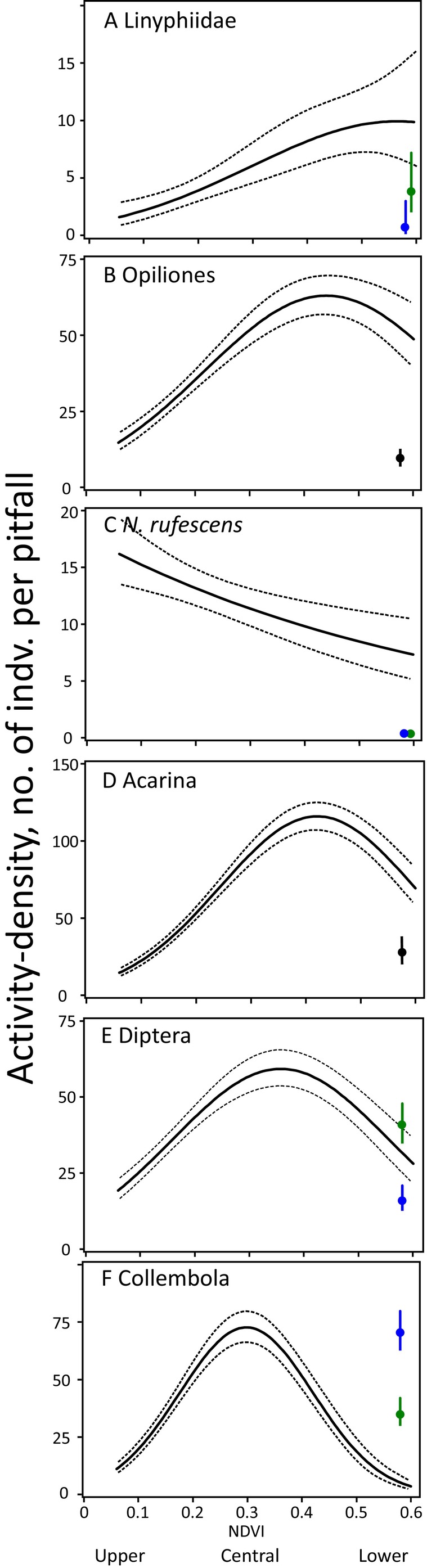
The relationship between the activity‐densities of Linyphiidae (A), Opiliones (harvestmen) (B), *N. rufescens* (adult ground beetles) (C), Acarina (mites) (D), Diptera (E), and Collembola (F) and the NDVI obtained by Poisson regression. Full line: Predicted mean model; 95% confidence bands: Dashed lines. Population means and 95% confidence limits from Kobbefjord where crowberry and gray willow habitats are shown as blue and green vertical lines.

NDVI values had varied effects on the activity‐densities of selected arthropod groups (Figure 3). The arthropod activity‐density response to NDVI was fitted to a second‐degree polynomial (Figure 3A–F). The ground beetles declined significantly from the low NDVI of the upper area to the high NDVI of the lower transect from 16.1 [13–19] to 7.4 [5–10] according to the linear Poisson regression model. Among prey, there was a significant hump‐shaped effect on activity‐densities of mites (Figure 3D), dipterans (Figure 3E), and collembolans (Figure 3F).

The activity‐density increase of the arthropods in relation to the vegetation development away from the glacier as well as in relation to the time since deglaciation leads to an arthropod population optimum 30–100 m away from the permanently snow‐covered area and 100–300 years after deglaciation. Further away from the permanently snow‐covered area and longer after deglaciation—where the vegetation has developed further—the arthropod activity‐density decreases. This pattern of activity‐density increase, and further away from the permanently snow‐covered area, decrease, was the situation for the potential prey, collembolans, dipterans, and mites as well as their potential arachnid predators, spiders, and harvestmen. The ground beetles showed steadily decreasing activity‐densities in relation to the developing vegetation cover away from the permanently snow‐covered area. The activity‐density of the potential prey showed early peaks in relation to the vegetation development while the spiders and harvestmen showed later peak trap catches in relation to the vegetation development.

The potential prey, springtails, showed the earliest trap catch peak in relation to the vegetation development. The mites may be both prey and predators which may explain the timing of their late trap catch peak.

The activity‐densities of *I. anglicana* in the glacier foreland in the summer of 2015 was 68% of all collembolans while activity‐densities in the summer of 2016 was 89% of all collembolans.

The activity‐density of *I. anglicana* was very different between the summer of 2015 and 2016 at the Qassinnguit glacier foreland area. The activity‐density in the summer 2016 was reduced by 82% compared to the average activity‐density in the summer 2015. Paired samples t‐test showed a highly significant difference (*p* ≤ 0.001; *N* = 14) in the *I. anglicana* activity‐densities between the two summer periods.

**FIGURE 4:**
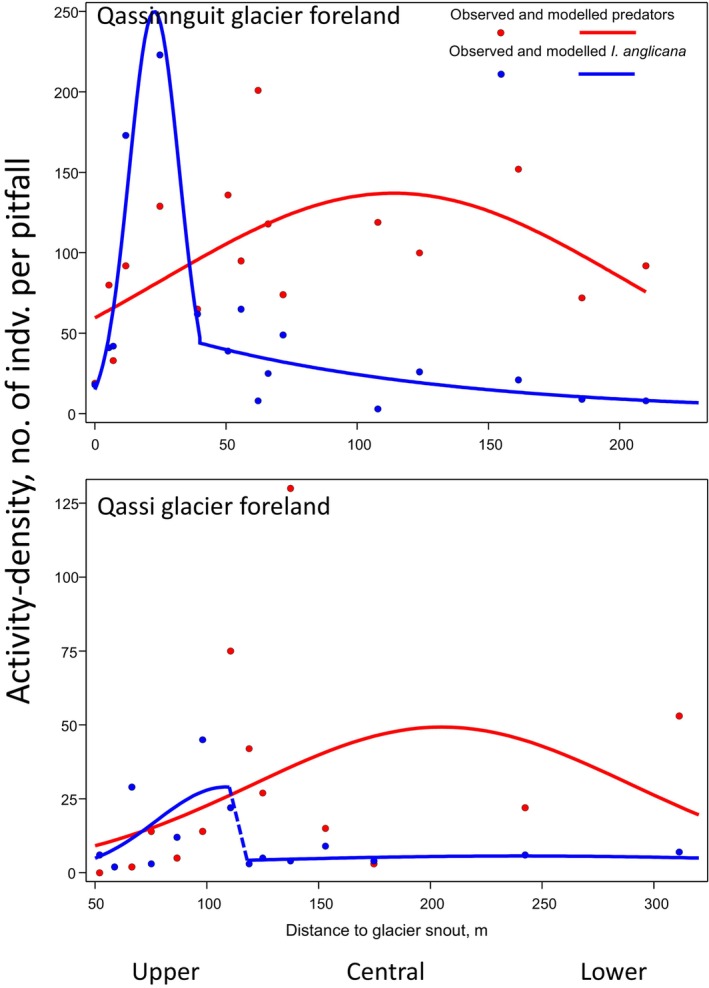
Activity‐density models obtained by Poisson regression of *I. anglicana* and the sum of potential arthropod predators (spiders, harvestmen, ground beetles, and mites) in vegetated plots in relation to the distance to the glacier snouts at the Qassinnguit, 2016, and Qassi, 2014, glacier forelands. *I. anglicana* curves represent the upper transect areas away from the glacier snout as well as a decline, further away.

Figure 4 shows activity‐densities of the dominant collembolan species *I. anglicana* and activity‐densities of the sum of potential arthropod predators—spiders, harvestmen, ground beetles, and mites—in relation to the distance to permanently snow‐covered area using data sampled in the summer of 2016 at the Qassinnguit glacier foreland and data sampled in the summer of 2014 at the Qassi glacier foreland.

**FIGURE 5:**
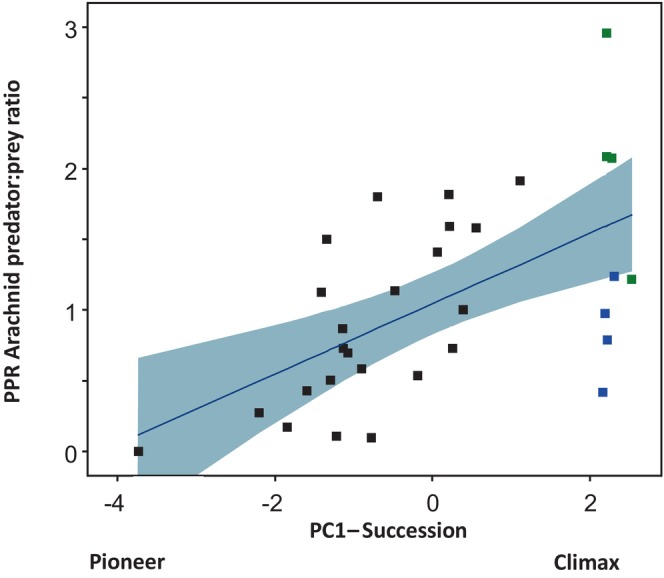
Linear regression of predator‐to‐prey ratios (*Y*‐axis) in relation to the Qassinnguit vegetation succession (*X*‐axis), with the vegetation development axis extended to include climax vegetation areas in the Kobbefjord Valley. The vegetation succession is represented by the first principal component (PC1) of a PCA ordination, based on NDVI, time since deglaciation, and distance to permanent snow cover (Table A3). The PC1 axis accounts for 90% of the variation in the data. “Arachnid Predator” abundance is defined as the sum of activity‐densities for Araneae (spiders) and Opiliones (harvestmen), while “prey” abundance is defined as the sum of activity‐densities for Collembola, Diptera, and Aphidoidea. Black square symbols represent pioneer vegetation in Qassinnguit, and blue and green squares represent crowberry and gray willow habitats in the Kobbefjord Valley. Linear regression results: *F* = 14.6, *p* < 0.001, with 95% confidence bands surrounding the regression line.

Figure 5 shows that the arachnid predator: Prey ratio increased from pioneer toward climax vegetation (linear regression *p* < 0.1%). The increase of this ratio is an indication of a shift from bottom‐up in the early succession stage—with a dominance of prey—while in the latter stage of succession—with a dominance of arachnid predators—top‐down mechanisms prevail.

### 3.3 Trophic Interactions—Hypothesis 3

**FIGURE A11:**
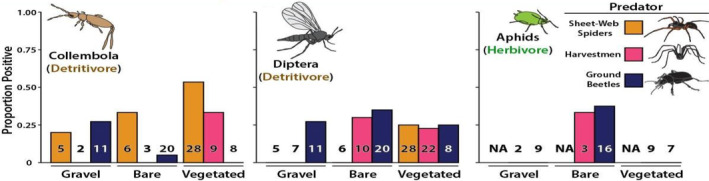
The proportion of collembolan, dipteran, and aphid prey in the guts of linyphiid spiders, *M. morio* (harvestmen), and Carabidae (ground beetles). Predators were collected in the summer of 2015 in the three different patch types—gravel, bare soil, and vegetated patches. Numbers on bars indicate the numbers of the three predators analyzed for gut contents. NA, Not applicable.

Figure A11 shows that the linyphiid gut contents of collembolans is closely related to the activity‐densities of collembolans in the three different habitats (gravel, bare, and vegetated). The figure also shows exceptional high gut contents of aphids in harvestmen and ground beetles caught in bare soil habitats where the activity‐densities of aphids were extremely low.

**FIGURE 6:**
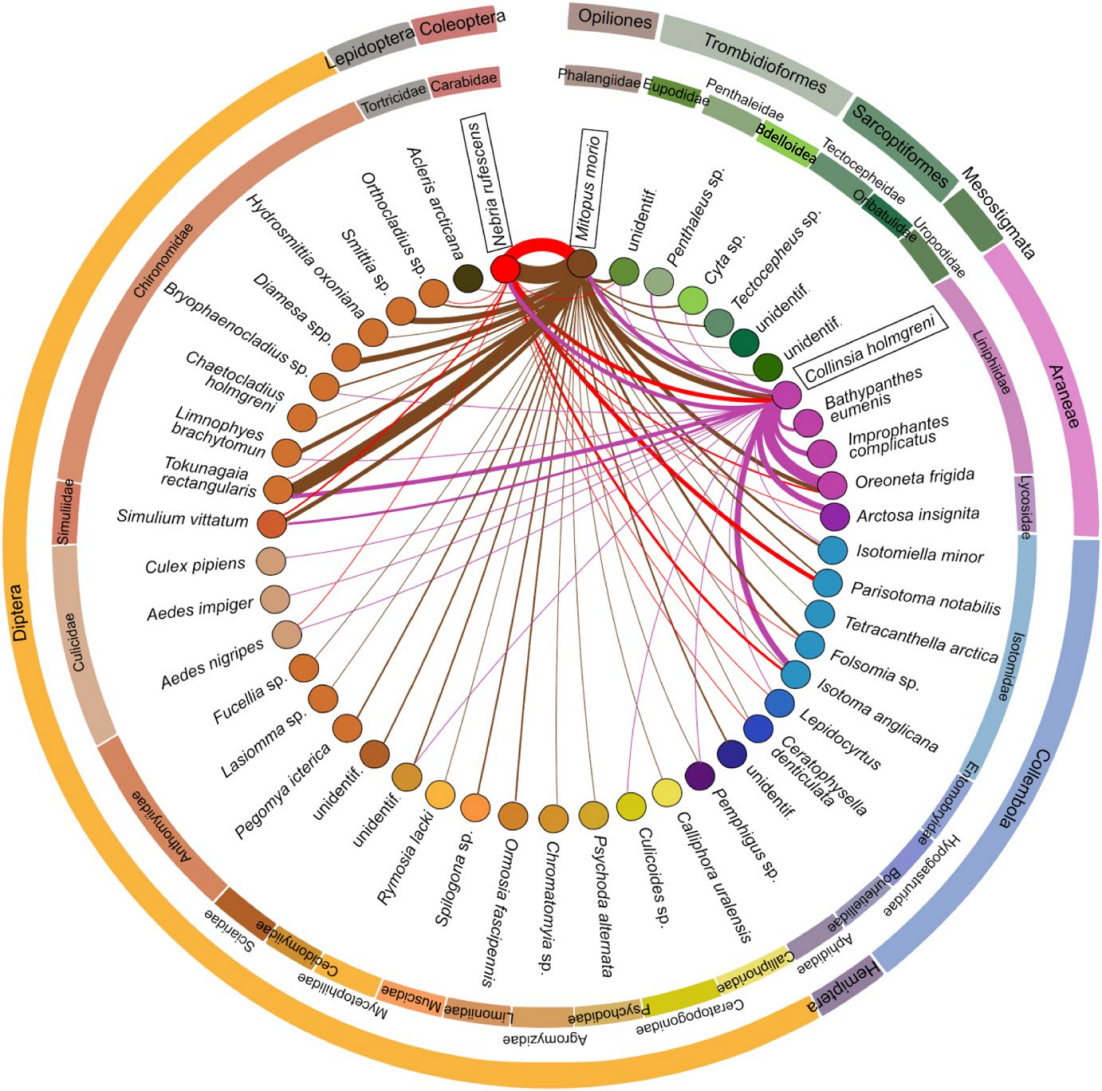
Food web construction based on gut content analysis. The studied predators are highlighted with black frames. The width of the links corresponds to the frequency of the observed interaction, representing the number of predators with the prey species identified by DNA metabarcoding in their gut. Red lines show links between *N. rufescens* and its prey, brown lines show links between *M. morio* and its prey, and purple lines show links between *C. holmgreni* and its prey. The outer ring categorizes both predators and prey into their respective taxonomic groups for additional context.

In 2016, the three predator species were analyzed using next generation sequencing (NGS). We obtained 12,786,590 reads as the total sequencing output. After processing (excluding erroneous reads and singletons), 12,650,364 reads remained as informative. The sequences were clustered into 130 OTUs (Operational Taxonomic Units) representing 11 orders. Hence, 30 families, 39 genera, and 44 species have been identified (Figure 6). The proportion of prey reads within each predator varied from 0% to 0.01% in ground beetles, from 0.12% to 66.5% in spiders ( *C. holmgreni* ) and from 0% to 10.0% in harvestmen. The average numbers of different prey species (OTUs) detected in a single predator were 1.10 (±1.0 SD) in adult ground beetles (0.56 ± 0.51 in their larvae), 4.9 (±1.67) in spiders, and 4.2 (±2.8) in harvestmen. In general, we found fewer prey species in the guts of predators collected in bare soil patches compared to those from vegetated patches.

The detailed gut content analysis revealed that the linyphiid spider had 22 different prey species (OTUs) in their guts, with *Oreoneta frigida* Thorell 1872 (Araneae: Linyphiidae) as the most common prey species (94%) of *C. holmgreni* sampled in the vegetated patches while Isotomidae collembolans were found in 50% of *C. holmgreni* from these patches. A lycosid spider, *Arctosa insignita* Thorell 1872, was found in 56% of the screened *C. holmgreni* and in the same number of the screened spiders chironomid flies were found as their prey. *Tokunagaia rectangularis* Goetghebuer 1940 was the most frequently found chironomid species found in 37.5% of the *C. holmgreni* .

Only one *C. holmgreni* individual was captured in the dry pitfall traps in the bare soil patches in the summer of 2016. This individual contained sequences of the harvestman, the ground beetle, and two linyphiid species.

*C. holmgreni* consumption of collembolans across all patch types was similar in the two summer periods with 46.2% in 2015 and 47.1% in 2016 even though collembolans—mainly *I. anglicana*—had a much higher activity‐density in the summer of 2015 compared to the summer of 2016.

The gut content analysis of the harvestman revealed 37 different prey species (OTUs). The ground beetles were the most common prey (87.5%) found in the harvestmen in bare soil patches. Seventy‐five percent of the harvestmen had chironomids in their guts in the bare soil patches and 68.8% in the vegetated patches. Linyphiid spiders and isotomids were found in 50% and 44% of the harvestmen sampled in the vegetated patches, respectively, while in bare soil patches, < 20% of those prey types were found in the harvestmen guts.

We found 14 different prey species (OTUs) in the guts of the ground beetles. The harvestman was the most common prey found in the guts of 56% and 37.5% of the ground beetles sampled in the bare soil and vegetated patches, respectively. The second most common prey were Collembola with 25% of the ground beetles positive from vegetated patches and 18.8% from the bare soil patches.

#### 3.3.1 Frequencies of Predation in Relation to Activity‐Density

**FIGURE 7:**
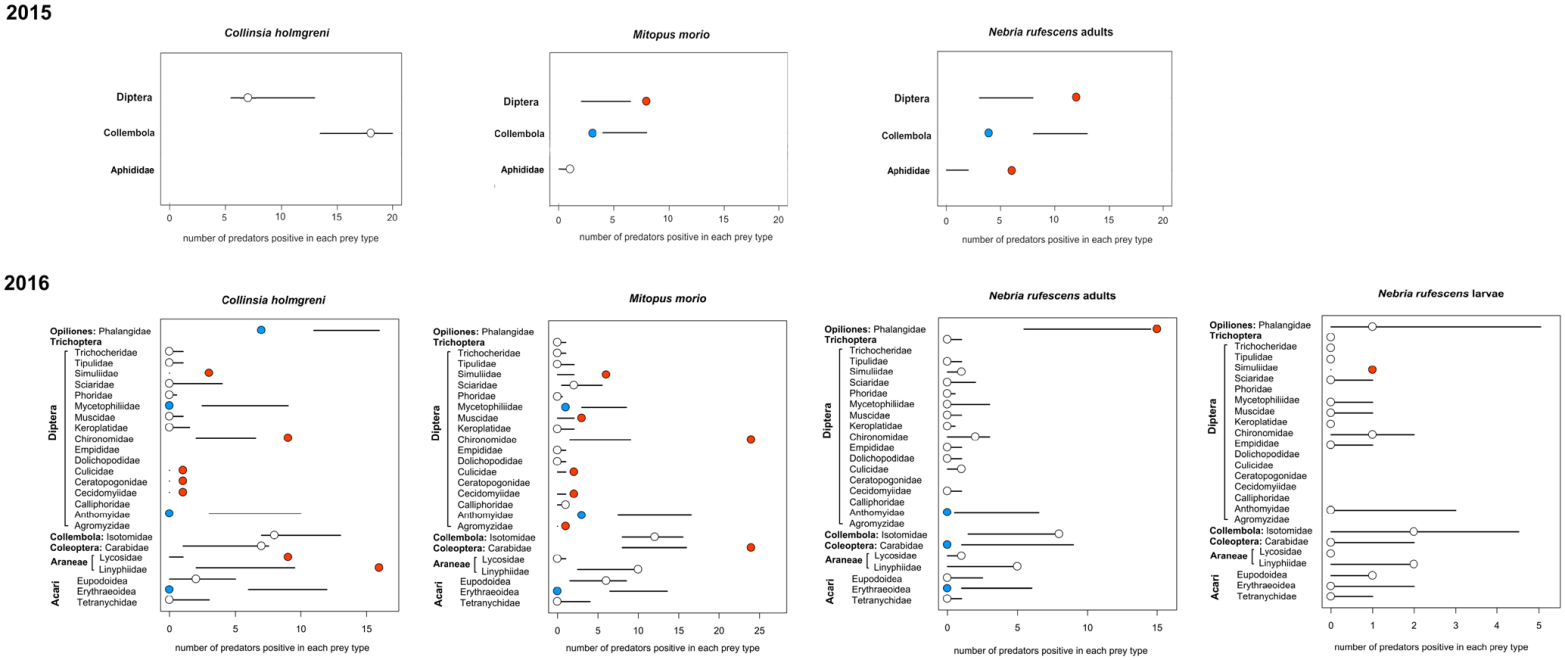
Food preferences of the predators sampled during the summers of 2015–2016 calculated from the gut content data and potential prey activity‐densities. The observed frequencies (dots) were compared to prey activity‐densities with the 95% CIs from the null model (horizontal lines). 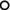 = frequency consistent with the null model (the observed predation frequency corresponded to the expected one based on prey activity‐density and gut content results); 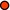 = frequency of predation was higher than expected in the null model; 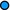 = frequency of predation was lower than expected according to the null model.

In 2015, ground beetles captured Diptera more frequently than expected, considering their activity‐density (AM5.2). In 2016, Diptera were less abundant at the sites, and their occurrence in the beetle guts aligned with predictions, as shown in Figure 7. Mainly adult beetles fed on harvestmen and collembolans more than expected from their activity‐densities. *C. holmgreni* selected other spiders and chironomids as prey and fed on harvestmen less than expected. Harvestmen fed on ground beetles and chironomids. Collembolans were exploited by all predators according to predictions based on their activity‐densities. Mites were underrepresented in the predator guts despite their abundance at the study site.

In 2016, Chironomidae represented the most exploited Diptera family, followed by Simuliidae, while Anthomyiidae, although the most abundant, were seldom found in the predator guts (Figure 7). Ninety percent of the anthomyiids were active in the late summer period which may have contributed to its low detection rate in predator guts.

#### 3.3.2 SEM Modeling

**FIGURE 8:**
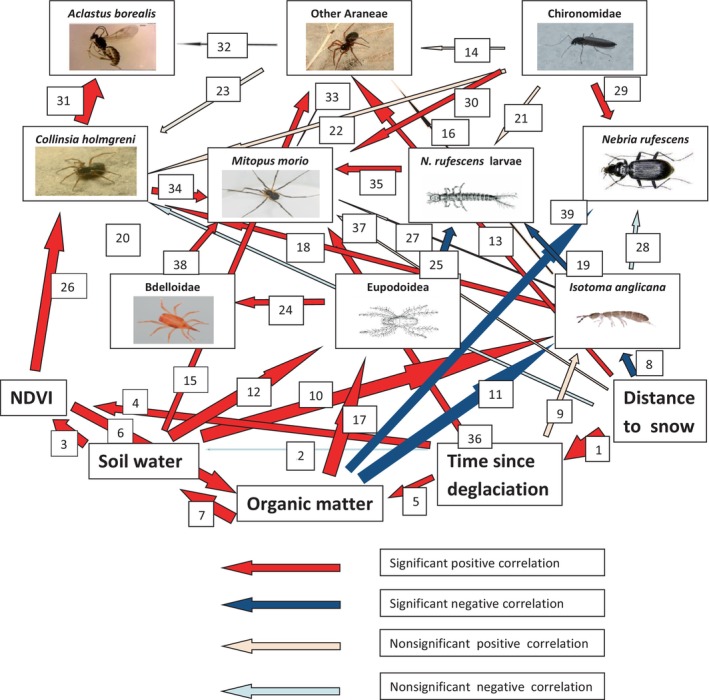
The path diagram presents a model, SEM1, for the relationships between activity‐densities of arthropod predators, activity‐densities of potential prey and important environmental variables using Structural Equation Modeling (SEM). Model fit: Chi‐square: 87.6; df: 66; Probability level: 0.039; Sample size: 44; CMIN/df: 1.327. For more information about measures of fit, see the Result section. The thickness of each arrow represents the standardized regression weights. Red arrows show significant positive correlations (*p* < 0.05) and blue arrows show significant negative correlations (*p* < 0.05). Pink arrows show nonsignificant positive correlations. Light blue arrows show nonsignificant negative correlation. The number at each arrow refers to more detailed information about each relationship in Table A6. See Acknowledgments for contributions by photographers.

The SEM1 diagram (Figure 8 and Table A6) shows the relationships between activity‐densities of the arthropod predators and their potential prey in relation to environmental variables. The fit of the best model had a Chi‐square of 87.6; df = 66; Probability level = 0.039; Sample size = 44; CMIN/df = 1.327; CFI = 0.942; RMSEA = 0.087; RMR = 0.104 (default model). These fit indices for the default model meet the recommended thresholds, indicating an acceptable fit to the data. This demonstrates that the data are robust and of high quality in supporting the SEM1 model.

The SEM1 model shows a positive feedback loop between soil water in the topsoil, NDVI, organic matter in the topsoil and back to soil water (for simple relationships see Table A3 and Figure A9). Overall, the result of the SEM1 analysis indicates that spiders, mites, and the harvestmen are all involved in bottom‐up food chains (positive correlations in the SEM1 diagram) while the ground beetle larvae are involved in top‐down food chains (negative correlations) with the collembolan species (*I. anglicana*) and Eupodoidea mites as potential prey (Table A6).

The relationship between the activity‐density of *I. anglicana* as potential prey and the activity‐density of *C. holmgreni* as potential predator was positively correlated indicating a bottom‐up food chain. The relationships between activity‐density of Chironomidae flies as prey and activity‐density of harvestmen and ground beetles as predators were also positively correlated, indicating bottom‐up food chains between this common dipteran family and these potential predators. The most abundant Diptera family in the glacier foreland—Chironomidae—was also the most common dipteran prey in the guts of all predators. Positive correlations were also found between activity‐densities of the arachnid predators, harvestmen, *C. holmgreni* spiders, Bdelloidea mites, and their prey, indicating bottom‐up food chains.

The relationships between activity‐densities of *I. anglicana*, and the adult ground beetle in relation to the organic matter content in the topsoil were all significant and negatively correlated.

Significant positive correlation was found between NDVI and the activity‐density of *C. holmgreni* . Significant positive correlations were also found between the activity‐density of other spiders and the distance to the glacier snout. The same was true for the activity‐density of harvestmen and the time since deglaciation, as time since deglaciation was closely correlated with the distance to the glacier snout.

### 3.4 Parasitism, IGP, and Destabilization of Linyphiid Populations—Hypothesis 4

The SEM1 diagram shows a highly significant positive correlation between the activity‐density of *C. holmgreni* and the activity‐density of the parasitic wasp, *Aclastus borealis* Boheman 1866 (Hymenoptera: Ichneumonidae).

**FIGURE 9:**
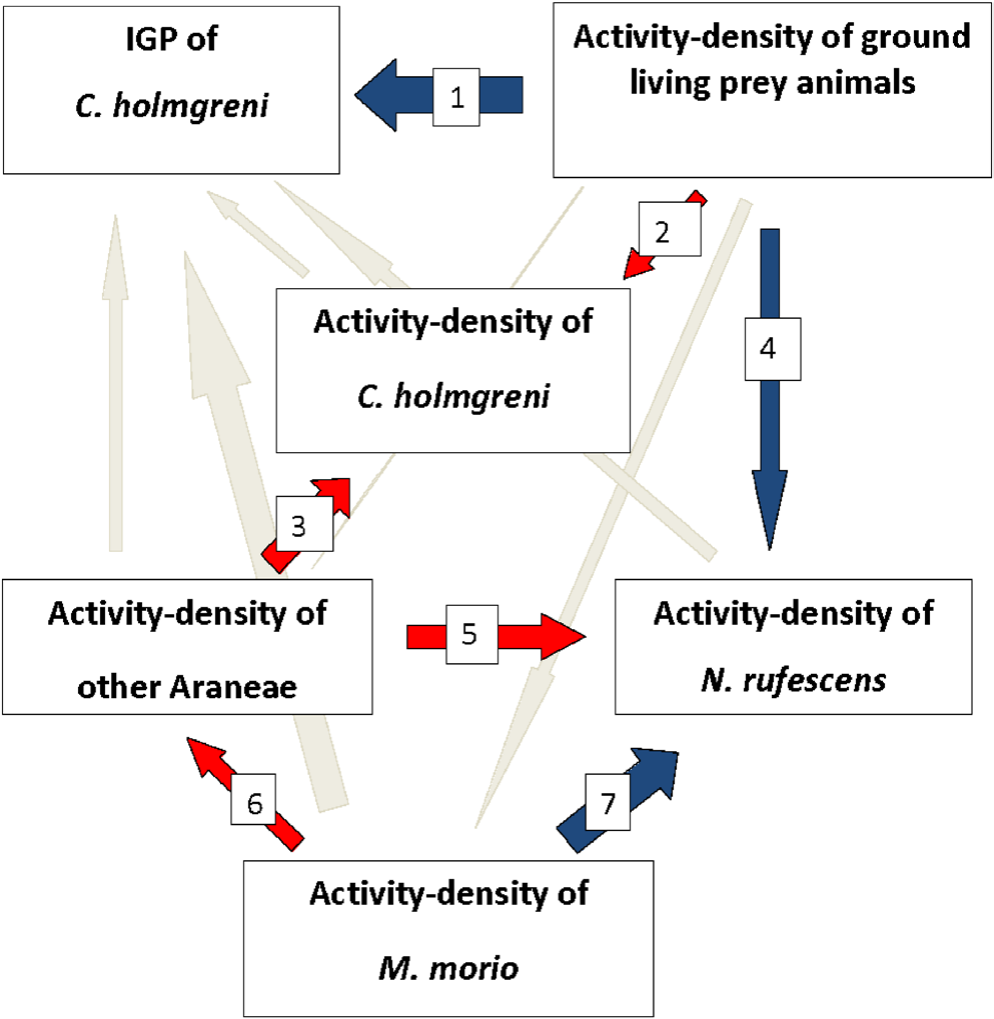
The path diagram presents SEM2, a model for the relationships between Intraguild Predation (IGP) of *C. holmgreni* , activity‐density of ground living preys, activity‐density of the ground beetles, activity‐density of the harvestmen, activity‐density of *C. holmgreni* as well as activity‐density of other Araneae using SEM. Model fit: Chi‐square = 0.021, df = 1; Probability level = 0.886; Sample size = 17; CMIN/df = 0.021; For more information about measures of fit, see the Result section. The thickness of each arrow represents the standardized regression weights. Red arrows show positive correlations and blue arrows show negative correlations. Gray arrows show nonsignificant correlations. The number at each arrow refers to more detailed information in relation to each correlation in Table A7.

Figure 9 shows the SEM2 diagram with the relationships between the IGP of *C. holmgreni* , activity‐density of ground‐dwelling preys (collembolans, mites, and aphids), activity‐density of *C. holmgreni* , activity‐density of other spiders than *C. holmgreni* , activity‐density of the ground beetles (adults and larvae), and activity‐density of harvestmen. The fit of the best model representing these relationships is: Chi‐square = 0.021; df = 1; Probability level = 0.886; Sample size = 17; CMIN/df = 0.021 (default model); CFI = 1; RMSEA = 0 (default model). In spite of the few replicates, this model has a good fit, and moreover, the model is supported by the DNA gut content results as well as the support of the literature for each of the shown trophic links (Table A7).

The relationship between the IGP of *C. holmgreni* and activity‐density of ground‐dwelling prey was highly significant, negatively correlated. The same was true for the relationship between the activity‐density of the ground beetles and the activity‐density of ground‐dwelling preys as well as the relationship between the activity‐density of the ground beetles and the activity‐density of harvestmen as both these relations were highly significant, negatively correlated.

The relationships between the activity‐density of *C. holmgreni* and the activity‐density of ground‐dwelling preys, the activity‐density of *C. holmgreni* and the activity‐density of other spiders as well as the activity‐density of the ground beetles and the activity‐density of other spiders were all highly significant, positively correlated. The relationship between the activity‐density of other spiders and the activity‐density of harvestmen were also significant, positively correlated.

During the summer of 2016, the research in the Qassinnguit glacier foreland showed that the average IGP‐ratio for *C. holmgreni* was 0.65 ± 0.25, *n* = 17. The average IGP‐ratio for the adult ground beetles was 0.44 ± 0.45, *n* = 32. The average IGP‐ratio for ground beetle larvae was 0.22 ± 0.49, *n* = 18. The average IGP‐ratio for the harvestmen was 0.38 ± 0.31, *n* = 32.

The average IGP‐ratio for *C. holmgreni* in relation to other linyphiid spiders was 0.33 ± 0.16, *n* = 17. The average IGP‐ratio for adult ground beetles in relation to linyphiid spiders was 0.09 ± 0.23, *n* = 32. The average IGP‐ratio for adult ground beetles in relation to the harvestmen was 0.34 ± 0.41, *n* = 32.

For further information regarding IGP, see Figure 9 and Table A7.

## 4 Discussion

### 4.1 Arthropod Populations in the Cold Glacier Foreland and in Warm Climax Habitats—Hypothesis 1

The dominance of linyphiid spiders in cold Arctic glacier forelands and lycosid spiders in warmer climax vegetation of Kobbefjord Valley is likely due to microclimate differences and disturbance levels (Bayram and Luff 1993; Buchs, Harenberg, and Zimmermann 1997; Basedow, Tóth, and Kiss 2000; Nyffeler and Sunderland 2003; Noel and Finch 2010; Birkhofer et al. 2018; Viel et al. 2022; Wehner et al. 2023). Disturbance plays a crucial role in predator distribution; linyphiids and ground beetles thrive in highly disturbed glacier forelands and early succession stages, while lycosids prevail in stable climax vegetation where there may be high lycosid IGP against linyphiids (Bowden et al. 2018) reducing the linyphiid abundances. Notably, the abundant Erigoninae subfamily member, *C. holmgreni* , thrives in disturbed habitats (Gravesen 2008; Freiberg et al. 2020).

#### 4.1.1 Arthropod Predators in the Glacier Foreland

Predator diversity in the Greenland glacier foreland is limited to a few species, primarily the ground beetle, *N. rufescens* , the harvestman, *M. morio* , and the spider *C. holmgreni* . Both the ground beetle and the harvestman are adapted to low temperatures and are common in South Western Greenland (Buse, Hadley, and Sparks 2001; Leirikh et al. 2009; Panagiotakopulu and Buchan 2015). The ground beetle thrives in bare soil habitats, consistent with prior findings (Gereben 1995).

*C. holmgreni* , a chionophilous spider, inhabits snow beds and late snow‐free areas in Western Greenland (Hein et al. 2014; Marusik 2015). It dominates after 40 years of deglaciation but is absent in warmer, climax‐vegetation areas like Kobbefjord, alongside six other linyphiid species exclusive to glacier forelands (Tables A8 and A9) (Pétillon, Courtial, and Vernon 2014; Hågvar, Ohlson, and Flø 2017). These spiders' ability to migrate, such as through ballooning, may help them adapt to changing climates (Coulson, Hodkinson, and Webb 2003b; Meijer 2008; Uma and Weiss 2010).

#### 4.1.2 Consequences of Warming

In the Qassinnguit glacier foreland, warming may have led to the extinction of cold‐adapted linyphiid spiders, likely due to prey scarcity and high intraguild predation (IGP) ratios destabilizing their populations. This aligns with Rall et al.'s (2010) findings that warming can decrease ingestion efficiencies in spiders and beetles, raising extinction risks as predator metabolic rates increase. Such metabolic changes may also impact linyphiid dominated habitats, like agricultural fields in climates where prey abundance may decline due to warming (Sohlström et al. 2022).

Additionally, Rall et al. (2010) suggest that warming might stabilize populations after small perturbations, such as the warm winter and spring of 2016, which initially reduced mite, collembolan, and dipteran populations but allowed recovery by summer. Although these conditions temporarily lowered spider activity‐densities, they did not lead to spider extinctions, unlike the effects of permanently warmer climates downhill. Dense climax vegetation in areas like the Kobbefjord Valley may buffer spiders from high IGP rates (Finke and Denno 2006), thus stabilizing populations compared to sparser pioneer habitats. In contrast, high predation in the gray willow habitat during the summer of 2016 diminished prey levels. The presence of a *C. holmgreni* individual with only intraguild prey DNA in the bare soil habitat in the glacier foreland suggests that IGP depends on vegetation complexity.

These findings highlight the complex impact of warming on predator–prey dynamics. As Rall et al. (2010) suggest, elevated temperatures can increase predator metabolic rates without a proportionate rise in ingestion rates, leading to reduced ingestion efficiencies. This metabolic imbalance could exacerbate prey scarcity in late‐succession habitats, as our SEM analysis indicates, where top‐down control may intensify due to increased predator energy demands. The observed shifts in predator–prey interactions from pioneer to climax habitats support the prediction that warming amplifies intraguild predation (IGP), particularly in sparsely vegetated areas where prey availability fluctuates.

#### 4.1.3 Consequences of Extreme Weather

Thick snow cover and few thaw periods in winter and spring 2015 (Table A1) likely protected predators like linyphiid spiders and their prey, such as collembolans, allowing them to survive under the snow (Coulson et al. 2000, 2023; Legault and Weiss 2013; Hågvar and Pedersen 2015; Greenland Ecosystem Monitoring 2024). Knowledge‐gaps exist regarding dipteran survival under extreme conditions, though they probably serve as prey for linyphiid and lycosid spiders and other arthropods in the snow (Agustí et al. 2003; Gratton, Donaldson, and Vander Zanden 2008; Chapman et al. 2013; Hambäck et al. 2016; Sanchez‐Ruiz et al. 2018). Even during warm years, snow affected by vegetation like gray willow, which acts as windbreaks, benefits arthropod predators by increasing local snowpack, insulating them from warm conditions in 2016.

#### 4.1.4 Population Developments Away From the Qassinnguit Glacier—Hypothesis 2

Population dynamics between arachnid predators and their potential prey are influenced by vegetation development stages, with bottom‐up mechanisms prevailing in early succession and top‐down control in later stages (Figures 3 and 5) (Bowyer et al. 2013). This shift is driven largely by increasing solar radiation which raises near‐surface temperatures away from the glacier (Høye and Forchhammer 2008a). NDVI is used to measure vegetation development and as an indicator of solar radiation levels. The shift from bottom‐up to top‐down is supported by the findings of Walker, Wilder, and González (2020) who showed increased killing rates by predatory spiders with warming.

Similar trends seen in Arctic succession research indicate an increase in spider populations as vegetation becomes more stable away from the glacier, suggesting a typical succession pattern (Denys and Tscharntke 2002; Coulson, Hodkinson, and Webb 2003b; Einarsdóttir 2010; Bråten et al. 2012). Early disturbances often trigger strong trophic cascades due to the amplified resource growth and susceptibility to predation, which initially boosts bottom‐up effects and later enhances top‐down effects as the system matures (Piovia‐Scott, Yang, and Wright 2017; Spiller, Schoener, and Piovia‐Scott 2018).

Close to the glacier snouts, high activity‐densities for *I. anglicana* (Figure 4), a high‐quality prey for ground beetles and spiders, suggest several influencing factors. These include both physiological adaptations and morphological traits like the furcula that give springtails an advantage in cold conditions, while predators may be less active and thus less predatory (Bauer 1982; Hågvar 2010; Sørensen and Holmstrup 2011; Roslin et al. 2017). As temperatures increase further from the glacier, nutrient release supports vegetation development, which in turn creates a litter layer enhancing the habitat for litter‐dwelling collembolans and mites. This increased litter density is shown to support higher densities of these organisms, with enhanced fungal activity in decomposing plant litter driving this trend (Hågvar and Klanderud 2009).

Further from snow‐covered areas, arthropod predators show higher activity‐densities, indicating a shift from bottom‐up to top‐down control mechanisms as indicated by the declining catches of *I. anglicana* due to increased predation. This supports the hypothesis that early succession stages are governed by resource availability, while predation becomes more influential in later stages.

Diurnal activity levels of dipterans are generally lower than those of their predators in cold conditions, impacting their ability to evade predation and explaining their frequent presence in the diets of predators (Kruse, Toft, and Sunderland 2008). This illustrates a temperature‐dependent evolutionary “arms race” between prey and predators, where different species may have advantages at varying points along the temperature gradient.

### 4.2 Trophic Linkages and Arthropod Food Web—Hypothesis 3

#### 4.2.1 Bottom‐Up and Top‐Down Mechanisms in Relation to Predators and Prey

In the summer of 2016, high search activity among ground beetles for scarce collembolans may have resulted in a strong top‐down effect on collembolan populations. This activity also have increased encounters with other arthropod predators, as evidenced by the contents of their guts, including spiders and harvestmen (Agustí et al. 2003; Harwood, Sunderland, and Symondson 2003; Marcussen, Axelsen, and Toft 2003) Linyphiid spiders, especially in low‐prey areas like gravel patches in summer 2015, exhibited active searching behavior for collembolans, indicating a flexible foraging strategy (Abrams 2010) This was evident from the DNA analysis showing consistent presence of Collembola DNA in linyphiid guts across both years, despite lower collembolan numbers in 2016.

Active searching by predators like linyphiids and ground beetles was also noted with rare aphids in 2015. Despite low activity‐density of aphids, they were frequently found in the guts of predators (Figure A11), suggesting deliberate targeting by these predators in areas devoid of alternative prey (Madsen, Terkildsen, and Toft 2004) In 2016, results were less clear due to technical issues with DNA amplification, but in 2015, a significant portion of beetles and harvestmen consumed aphids, mainly in bare soil areas where aphids were more exposed and easier to capture (Lang and Gsödl 2001) This behavior underscores a top‐down control effect, where predators intensify their search for available prey following depletion of more abundant species.

The evidence of strong top‐down effects on collembolan populations, as seen with active hunting behaviors, supports the hypothesized influence of hunting strategies on predator–prey dynamics.

#### 4.2.2 SEM Modeling

Our study utilized SEM to analyze arthropod feeding dynamics, focusing on the main feeding modes across various groups (Figure 8). We developed a general SEM model to evaluate the relationships among arthropod groups, their prey, and environmental variables. A more detailed model explored the intraguild predation (IGP) rates of *C. holmgreni* in relation to the activity‐density of available prey and other arthropod predators.

The general SEM model revealed that spiders, harvestmen, and adult ground beetles are primarily influenced by bottom‐up food chains connected to their potential prey populations. This model suggests that these predators remain in areas with high prey density, particularly when their prey includes chironomids, demonstrating clear bottom‐up effects (Sanchez‐Ruiz et al. 2018) The study's findings align with observations from temperate agricultural fields, indicating similar ecological interactions occur in diverse environments (Table A10, Figure A10) where Gravesen (2008) found that linyphiid spiders were dependent on bottom‐up food chains.

Conversely, the ground beetle larvae were engaged in distinct top‐down food chains, particularly with sedentary prey such as the Eupodoidea mites. The SEM results show significant negative correlations between these predators and their prey, indicating that active predators can reduce prey populations within localized patches. This relationship highlights the importance of predator mobility and prey sedentariness in shaping local ecosystem dynamics (Huey and Pianka 1981).

Furthermore, our study examined the ecological implications of high predation rates on key detritivores like mites and collembolans. These organisms play a critical role in the decomposition of organic matter, which contributes to soil structure and nutrient cycling. The SEM findings suggest that increased predation leads to reduced activity densities of these detritivores, subsequently resulting in higher organic matter content in the topsoil (Lawrence and Wise 2000, 2003; Liao et al. 2016). This has broader implications for carbon storage in Arctic soils, particularly under warming scenarios where increased organic matter can enhance soil's capacity to act as a carbon sink (Schmitz et al. 2014; Vilmundardóttir et al. 2018; Mankasingh and Gisladóttir 2019; Khedim et al. 2021).

The results of our SEM analysis not only corroborated several existing ecological hypotheses but also generated novel insights. Notably, the models identified positive correlations between the activity‐density of *C. holmgreni* and other spider species, suggesting that these spiders may be important intraguild prey for *C. holmgreni* (Cuff et al. 2021).

SEM results also illustrate how hunting strategies, particularly those of active hunters like ground beetle larvae, contribute to top‐down control, especially in localized patches with high prey density.

#### 4.2.3 Hunting Strategies and Trophic Linkages

Our findings offer insights into the trophic interactions and strategies highlighted in Hypothesis 3, where predator foraging methods play a pivotal role in shaping predator–prey dynamics. Gut content analysis identified that linyphiid spiders, which often employ sit‐and‐wait hunting strategies, rely more on prey density, especially in areas with limited alternative food sources. This dependence on local prey availability aligns with expectations that sit‐and‐wait predators are constrained by immediate resource accessibility, making them more sensitive to fluctuations in prey abundance.

In contrast, the gut contents of ground beetle larvae, characterized as active hunters, reflected a broader range of prey species. This finding supports the hypothesis that active predators exert more direct control over prey populations, actively seeking prey across a larger area. SEM analysis showed that this active hunting strategy contributes to localized top‐down control, especially for sedentary prey like Eupodoidea mites, where predator mobility enhances their capacity to reduce prey densities within specific patches.

These observations indicate that sit‐and‐wait and active hunting strategies yield distinct trophic impacts, with active hunters potentially exerting stronger top‐down effects in late‐succession habitats. As temperatures rise and alter habitat composition, these predator–prey dynamics are likely to shift, with hunting strategies playing a critical role in influencing prey population stability and ecosystem balance.

### 4.3 Parasitism and IGP Leading to Population Destabilization—Hypothesis 4

*C. holmgreni* faces threats from parasitoids like *A. borealis* , which parasitizes its egg sacs, especially when spider activity is high (Punzo and Ludwig 2005; Punzo 2006; Uma and Weiss 2010). *A. borealis* is not exclusive to *C. holmgreni* as it also occurs where this spider is absent (Coulson, Hodkinson, and Webb 2003a). Parasitism by *A. borealis* may significantly impact linyphiid populations during the summer (Wise 1993; Finch 2005).

#### 4.3.1 IGP Among *C. holmgreni* Because of Low Prey Availability

During the summer of 2016, low availability of collembolans and other prey may have increased intraguild predation (IGP) ratios among *C. holmgreni* . Forced to abandon low‐quality web‐sites, these linyphiid spiders likely increased their ground movements in search of scarce prey, leading to more frequent encounters with other arthropod predators, such as other linyphiids and lycosid spiders, thereby elevating IGP ratios (Wise 2006; Chapman et al. 2013). *C. holmgreni* , a member of the Erigoninae subfamily, may switch from web‐building to active hunting based on prey availability, resulting in higher IGP during periods of low prey capture.

The inability to detect cannibalism via metabarcoding suggests that actual IGP instances might be underestimated (Cuff et al. 2021). High IGP ratios observed in 2016 could be a temporary response to exceptionally low prey availability, indicating that these strong trophic cascades may occur during short periods (Piovia‐Scott, Yang, and Wright 2017).

#### 4.3.2 Linyphiid IGP Against Lycosids

In the Qassinnguit glacier foreland, we observed a notable decrease in lycosid numbers from 27 individuals in 2015 to just one in 2016, specifically *Pardosa groenlandica* . This drop occurred despite earlier snow melt in 2016, suggesting factors other than weather influenced these results. Lower prey availability in 2016, particularly of mites, collembolans, and dipterans, may have increased intraguild predation (IGP). Although pitfall traps primarily measure activity‐density and not presence, the data suggest significant predation dynamics. For instance, *Arctosa insignita* was detected in 2015 trap catches and was absent in 2016, yet it was commonly found in the diet of *C. holmgreni* , with over half of the captured *C. holmgreni* containing *A. insignita* DNA, indicating high IGP, particularly among juveniles of *A. insignita* . This high IGP rate between *C. holmgreni* and *A. insignita* suggests that linyphiid spiders can maintain dominance in the glacier foreland, especially during warmer periods.

#### 4.3.3 IGP of *C. holmgreni* in Relation to Potential Preys—and in Relation to Other Predators

The SEM model (Figure 9) shows that the IGP‐ratio of *C. holmgreni* is negatively correlated with the activity‐density of ground‐dwelling prey such as collembolans, aphids, and mites, aligning with findings by (Lucas and Rosenheim 2011). The model suggests that high activity‐density of ground beetles may reduce prey density, thus increasing IGP‐ratios of *C. holmgreni* . Positive correlations between the activity‐densities of *C. holmgreni* and other spiders, and between other spiders and harvestmen, indicate significant food chain interactions, suggesting that these spiders and harvestmen form an important food resource for *C. holmgreni* (Lucas and Maisonhaute 2019). The SEM model indicates that IGP is an important food source with significant correlations between each predator. This may suggest a vulnerable food web between the predators making the most vulnerable predators prone to extinction (Dawey et al. 2013).

The relationship between the activity‐density of ground beetles and other spiders is positive, while it is negative with harvestmen, indicating differential predation risks among these arthropods. Linyphiid spiders, which use webs for protection, are less vulnerable to predation by ground beetles compared to the more exposed harvestmen (Dawey et al. 2013). High IGP‐ratios were observed where 47% of ground beetles had harvestmen in their guts, contrasting with only 16% having linyphiid DNA, reflecting varied intraguild predation pressures (Raso et al. 2014).

Food scarcity leads to intraguild predation among arthropod predators, especially affecting juveniles (Polis, Myers, and Holt 1989; Hodge and Marshall 1996; Montserrat et al. 2012). Research has shown reciprocal intraguild predation impacts on mite population developments, with some species going extinct under certain ratios (Marques et al. 2018). This dynamic could similarly affect arthropod predators in glacier forelands, with populations of common species like *C. holmgreni* and ground beetles potentially declining as vegetation progresses to a climax succession stage, while harvestmen persist due to better antipredatory adaptations (Chelini, Willemart, and Hebets 2009; Dias and Willemart 2013; Garcia‐Hernández and Machado 2017).

The IGP‐ratio among *C. holmgreni* , particularly against other spider species, is notably high (IGP‐ratio: 0.46 ± 0.18, *n* = 17). This suggests that anti‐intraguild predation behavior may have evolved in environments with low prey availability, posing a constant threat to juveniles (Walzer and Schausberger 2011, 2012). Antipredatory behavior could help stabilizing linyphiid populations under high IGP and parasitism pressures (Wise 2006). A SEM model analysis in experimental Danish wheat fields shows a negative correlation between linyphiid web density and juvenile production, indicating that linyphiid females may locate their egg sacs as far away from high web densities to avoid predation on egg sacs and juveniles (Figure A3) (Van Baarlen, Sunderland, and Topping 1994; Gravesen 2008). Such behaviors could help stabilize linyphiid populations under high IGP and parasitism pressures (Wise 2006). Such antipredatory behavior may also exist in the glacier foreland where it may stabilize the linyphiid populations.

## 5 Conclusion

The most significant finding of the study is that cold‐adapted linyphiid populations may destabilize and may go extinct as the microclimate warms, moving away from the glacier. This destabilization in the glacier foreland may be a consequence of low prey availability which may lead to high IGP among all the arthropod predators—particularly among the linyphiid spider species. The background for the high IGP among linyphiid spiders may be a shift in predation modes from a sit‐and‐wait behavior to more active search behavior. Additionally, the increased predation pressure may have influenced the development of antipredatory behaviors among IG prey species.

The SEM modeling shows that the arachnid predators—spiders, harvestmen, and bdelloid mites—are dependent on bottom‐up food chains while the ground beetle larvae are dependent on top‐down food chains. These mechanisms may be closely related to the hunting strategies of the predators as a bottom‐up mechanism may be connected to sit‐and‐wait behavior while top‐down mechanism may be related to active‐search behavior. The SEM modeling also shows that spider egg parasitism by *A. borealis* may be important and may have negative influence on the linyphiid population developments in the glacier foreland.

Heavy predation on the detritivores (collembolans and mites) have consequences for the organic matter content in the topsoil as declining activity‐densities of the detritivores are responsible for the increase in the organic matter content of the topsoil.

## Author Contributions

**Ejgil Gravesen:** conceptualization (lead), formal analysis (equal), investigation (lead), methodology (lead), writing – original draft (lead), writing – review and editing (equal). **Lenka Dušátková:** conceptualization (equal), data curation (equal), formal analysis (equal), investigation (equal), methodology (equal), supervision (equal), validation (equal), visualization (equal), writing – original draft (equal), writing – review and editing (equal). **Kacie J. Athey:** conceptualization (equal), data curation (equal), formal analysis (equal), funding acquisition (equal), methodology (equal), writing – original draft (equal), writing – review and editing (equal). **Jiayi Qin:** conceptualization (equal), data curation (equal), formal analysis (equal), investigation (equal), methodology (equal), software (equal), writing – original draft (equal), writing – review and editing (equal). **Paul Henning Krogh:** conceptualization (lead), data curation (lead), formal analysis (lead), funding acquisition (lead), investigation (supporting), methodology (supporting), project administration (equal), resources (equal), software (lead), supervision (equal), validation (lead), visualization (supporting), writing – original draft (equal), writing – review and editing (lead).

## Conflicts of Interest

The authors declare no conflicts of interest.

## Data Availability

Activity‐density population data, gut content arthropods, and environmental measurements are provided in the Dryad repository via the link: https://doi.org/10.5061/dryad.qfttdz0qt. Raw amplicon sequence reads from gut content analysis have been deposited in the NCBI BioProject under accession numbers PRJNA1188582.

## Appendix 1

**TABLE A1:** Detailed climatic data. The research conducted between 2015 and 2017 at the Qassinnguit and Qassi glacier forelands and the climax vegetation areas in Kobbefjord Valley focused on the climatic differences between these years. This table indicates notable differences in climate data from Kobbefjord Valley for the years 2015 and 2016. Specifically, the mean air temperature during the first half of 2015 was significantly lower than during the same period in 2016. Additionally, the winter and spring of 2015 had more snow cover and fewer thaw periods compared to 2016, resulting in longer lasting snow cover, particularly at the Qassinnguit glacier foreland. These climatic variations are essential for understanding seasonal and annual changes in these environments.

	2015	2016	2017
Kobbefjord Valley climate data^a^			
Mean air temp. January 1–June 30	−5.73°C	−0.29°C	−3.34°C
Mean air temp. July	10.2°C	10.6°C	9.59°C
Number of thaw periods January 1–April 30.	9	25	14
Total precipitation June 1–August 15.	52.8 mm	44.6 mm	150.5 mm
Mean snow cover from January 1.	86 cm until June 12	23 cm until April 27	80 cm until June 4
Time free of snow cover^b^			
Qassinnguit glacier foreland	July 1	May 14	June 28
Crowberry site	June 15	April 20	June 1
Gray willow site	June 19	April 24	June 5

^a^ Greenland Ecosystem Monitoring (2020c).

^b^ ASIAQ Greenland Survey (2023) and Sinergise Ltd. (2023).

**TABLE A2:** Patch labels, NDVI, distance to glacier, and lichen diameter. Patch labels, patch type, NDVI, distance to glacier snout, lichen mean diameter (recorded in 2016), and age of deglaciation. See AM2 for detailed information on lichenometric dating and age estimation.

	Patch label	Patch type	NDVI	Distance to glacier snout m	Mean lichen diameter mm	Age years
Upper transect	1	Bare soil	0.063	0	0	0
	2	Gravel	0.084	5.35	8.3	38
	3	Vegetated	0.13	6.95	12.3	72
	4	Vegetated	0.242	11.7	16.2	145
	5	Vegetated	0.276	24.75	19.6	262
Central transect	A	Gravel	0.099^a^	99.3	25.4	630
	B	Gravel	0.155^a^	91.65	26.0	654
	C	Gravel	0.136^a^	67.35	12.0	70
	D	Gravel	0.146^a^	58.45	14.5	105
	E	Gravel	0.109^a^	50.65	18.6	224
	F	Gravel	0.144^a^	48.65	13.8	95
	G	Gravel	0.213^a^	42.85	14.5	105
	I	Vegetated	0.215	107.8	55.5	2538
	J	Vegetated	0.391	71.7	18.8	228
	K	Vegetated	0.595	66	23.7	510
	L	Vegetated	0.524	62.15	20.4	309
	M	Vegetated	0.248	55.7	13.3	85
	N	Vegetated	0.142	39	13.6	90
	O	Vegetated	0.391	50.7	14.8	106
	P	Bare soil	0.125	109.75	10.9	60
	Q	Bare soil	0.122	79.8	23.6	500
	R	Bare soil	0.105	70.1	15.5	120
	S	Bare soil	0.135	66.7	15.0	117
	T	Bare soil	0.115	67.8	9.7	49
	U	Bare soil	0.101	53.7	10.8	58
	V	Bare soil	0.079	43.1	9.3	45
Lower transect	6	Vegetated	0.335	123.7	36.0	1269
	7	Vegetated	0.329	161.4	28.6	821
	8	Vegetated	0.327	185.6	38.0	1472
	9	Vegetated	0.543	210	48.3	1974

^a^ Age of deglaciation estimated from soil water, organic matter, and age.

**TABLE A3:** Environmental correlation matrix. Pearson correlation coefficients, *n* = 44 (NDVI *n* = 23) and level of significance for environmental variables. Bold numbers are significant correlations.

	Distance to glacier snout	Soil water	Organic matter	Time since deglaciation	NDVI
Distance to glacier snout					
				
Soil water	4%				
	80%				
Organic matter	**44%**	**74%**			
	0.3%	< 0.01%			
Time since deglaciation	**67%**	**32%**	**62%**		
	< 0.01%	3%	< 0.01%		
NDVI	**46%**	**76%**	**82%**	**61%**	
	3%	< 0.01%	< 0.01%	0.2%	
Meter above sea level (MASL)	**−98%**	7%	**−36%**	**−60%**	−36%
	< 0.01%	63%	2%	< 0.01%	9%

*Note:* This correlation matrix is symmetrical with same values above and below the gray shades cells, as correlations between two variables are identical regardless of their order. To avoid redundancy, only the lower half of the matrix is presented. The grey‐shaded diagonal indicates self‐correlations (100%), which are trivial and not meaningful for interpretation. It does not indicate significance. Significance is shown by bold numbers and %‐ages below.

**TABLE A4:** Taxons and barcode references. Taxon name list of collected specimen in 2015 and 2016 along with the BOLD barcode index number (BIN) and the BOLD process ID or the GenBank accession number for COI barcoded specimen, see AM5.1. Species without a process ID has been identified by morphologic characters.

Order	Suborder/family	Species	Process ID
Coleoptera	Carabidae	*Nebria rufescens*	SBS‐249
			SBS‐250
Opiliones	Phalangiidae	*Mitopus morio*	
Araneae	Linyphiidae	*Collinsia holmgreni*	GREAR010‐17
		*Oreoneta frigida*	GREAR016‐17
		*Improphantes complicatus*	GREAR091‐17
		*Bathyphantes eumenis*	GREAR008‐17
			GREAR009‐17
		*Oreonetides vaginatus*	GREAR017‐17
			GREAR088‐17
		*Islandiana princeps*	GREAR012‐17
		*Walckenaeria karpinskii*	GREAR020‐17
			GREAR094‐17
		*Walckenaeria clavicornis*	GREAR019‐17
		*Agyneta nigripes*	GREAR007‐17
		*Mecynargus borealis*	
		*Mecynargus paetulus*	GREAR015‐17
		*Erigone arctica*	
		*Centromerus arcanus*	
		*Agyneta jacksoni*	GREAR006‐17
	Lycosidae	*Pardosa groenlandica*	
		*Arctosa insignita*	GREAR021‐17
			GREAR095‐17
		*Pardosa hyperborea*	GREAR024‐17
	Thomisidae	*Xysticus durus*	GREAR029‐17
	Hahniidae	*Hahnia glacialis*	GREAR005‐17
	Philodromidae	*Thanatus arcticus*	
Collembola	Isotomidae	*Isotoma anglicana*	No barcode
Diptera	Chironomidae		
	Sciaridae	BOLD:AAM9258 *Camptochaeta consimilis*	GREAR134‐17
		BOLD:AAM9258 *Camptochaeta consimilis*	GREAR139‐17
		BOLD:ACK8050 Sciarid No species name	GREAR140‐17
	Anthomyiidae	BOLD:AAG2437 *Fucellia tergina*	GREAR150‐17
		BOLD:AAG2437 *Fucellia ariciiformis*	GREAR152‐17
		BOLD:AAG2477 *Pegomya icterica*	GREAR153‐17
		BOLD:AAG2437 *Fucellia tergina*	GREAR149‐17
		BOLD:AAG2437 *Fucellia tergina*	GREAR150‐17
		BOLD:AAG2437 *Fucellia tergina*	GREAR152‐17
		BOLD:AAG2477 *Pegomya icterica*	GREAR153‐17
		BOLD:AAG2437 *Fucellia tergina*	GREAR154‐17
		BOLD:AAG2437 *Fucellia tergina*	GREAR155‐17
	Mycetophilidae	BOLD:AAG4872 *Mycetophila perpallida*	GREAR118‐17
			GREAR119‐17
		BOLD:AAM9014 *Exechia frigida*	GREAR121‐17
		BOLD:AAM9014 *Exechia frigida*	GREAR122‐17
		BOLD:AAM9014 *Exechia frigida*	GREAR123‐17
		BOLD:AAM9014 *Exechia frigida*	GREAR125‐17
		*Mycomya sp*.	GREAR126‐17
	Muscidae	BOLD:AAP9046 *Spilogona megastoma*	GREAR158‐17
		BOLD:AAM9109 *Spilogona sanctipauli*	GREAR159‐17
		BOLD:AAM9109 *Spilogona sanctipauli*	GREAR160‐17
		BOLD:ACF0384 *Spilogona arctica*	GREAR161‐17
		BOLD:ACG4892 *Spilogona alpica*	GREAR162‐17
	Simuliidae	BOLD:AAA4121 *Simulium vittatum*	GREAR115‐17
	Culicidae	BOLD:AAA3750 *Aedes impiger*	GREAR112‐17
		BOLD:AAA3750 *Aedes impiger*	GREAR113‐17
	Empididae	BOLD:ACA4410 *Rhamphomyia hirtula*	GREAR141‐17
		4 replicates	GREAR142‐17
			GREAR143‐17
			GREAR144‐17
	Cecidomyiidae		
	Tipulidae		
	Phoridae		
	Dolichopodidae		
	Agromyzidae	BOLD:ACA8845 *Chromatomyia puccinelliae*	GREAR148‐17
	Scatophagidae	BOLD:ADY1455 *Scathophaga furcata*	GREAR163‐17
	Ceratopogonidae		
	Calliphoridae		
	Keroplatidae		
	Trichoceridae		
Trichoptera			
Hemiptera	Aphididae	BOLD:AAB2729 *Thecabius populimonilis*	SBS‐190 SBS‐191
Acarina	Erythraeoidea		
	Eupodoidea		
	Bdellidae		
	Tetranychidae	*Bryobia* sp.	
Mesostigmata	Gamasida		
Hymenoptera	Ichneumonidae	*Aclastus borealis*	
		BOLD:AAA9140 *Aclastus sp*.	GREAR172‐18
		BOLD:AAA9140 *Aclastus sp*.	GREAR174‐18
		BOLD:ACA4244 *Barycnemis bellator*	GREAR186‐18
		BOLD:AAF4291 *Plectiscidea moerens*	GREAR187‐18
		BOLD:AAF1390 *Phygadeuon* sp.	GREAR179‐18
		*Orthocentrini* sp.	
		*Plectiscidea sp*.	GREAR187‐18
	Braconidae	BOLD:ACA4555 *Microplitis coactus*	GREAR184‐18 GREAR185‐18
	Pteromalidae	*Ardilea convexa*	GREAR165‐17
	Eulophidae	*Hemiptarsenus unguicellus*	GREAR166‐17
		*Chrysocharis cf. pubicornis*	GREAR167‐17
	Diapriidae	*Zygota ruficornis*	GREAR168‐17

**TABLE A5:** Diversity indices. Mean species richness, *S*, and Shannon diversity, *H* = −∑ *p*_*i*_ log_2_ *p*_*i*_, for all arthropods and separately for prey and predators. Small letters, a, b, and c, indicate significant differences, Tukeys's *p* < 5%.

			All		Prey		Predators	
			S	H	S	H	S	H
2015	Bare soil/gravel	Central	12.7^b^	2.4	11.7^b^	2.3	4.9^c^	1.6^bc^
	Vegetated		19.9^a^	2.8	16.4^a^	2.8	10.7^a^	2.4^a^
2016	Bare soil/gravel	Upper	11.0^b^	2.3	10.5^b^	2.4	3.5^c^	0.8^c^
		Central	11.9^b^	2.7	10.9^b^	2.7	5.4^bc^	1.7^bc^
	Vegetated	Upper	12.0^b^	2.3	11.0^b^	2.2	5.3^bc^	1.6^bc^
		Central	13.9^b^	2.8	12.1^b^	2.8	6.9^b^	1.8^b^
		Lower	14.8	2.8	12.3^ab^	2.7	7.5^b^	1.8^abc^

**TABLE A6:** SEM1 interaction pathways between environmental variables and taxons and between taxons. Pathways (Figure 8), standardized regression weights, significance levels, the % gut content represents the percentage of predators that have a specific prey species in their gut out of the total number of individuals examined and literature references supporting the presumed process. The type of relationship in the SEM model is shown by → for unidirectional causation.

No. of pathway	Pathway	Standardized regression weights and significance levels	Gut content % in vegetation/bare soil habitats	References
1	DS → TSD	0.69***		See Table A2
2	TSD → SW	−0.06 ns		Wojcik et al. (2020), see Table A2
3	SW → NDVI	0.53***		Liu et al. (2012), Ahmed et al. (2017), see Table A2
4	TSD → NDVI	0.39***		Schütte et al. (2009), see Table A2
5	TSD → OM	0.32**		Wojcik et al. (2020) Vilmundardóttir et al. (2018), see Table A2
6	NDVI → OM	0.53***		Mankasingh and Gisladóttir (2019), Pérez (2001) see Table A2
7	OM → SW	0.63***		Védère et al. (2022), see Table A2
8	DS → *I. anglicana*	−0.38*		Hågvar and Gobbi (2022)
9	TSD → *I. anglicana*	0.29		Hågvar and Gobbi (2022)
10	SW → *I. anglicana*	0.69***		Liu et al. (2015), Liao et al. (2016)
11	OM → *I. anglicana*	0.63**		Lawrence and Wise (2000, 2003), Liu et al. (2015), Liao et al. (2016)
12	SW → Eupodoidea	0.46*		Liu et al. (2015), Liao et al. (2016)
13	DS → Other Araneae	0.45***		Bråten et al. (2012), Bowden et al. (2015)
14	Chironomidae → Other Araneae	0.11 ns		Gratton, Donaldson, and Vander Zanden (2008), Hambäck et al. (2016), Sanchez‐Ruiz et al. (2018)
15	SW → Other Araneae	0.43***		Allen et al. (2014), Liu et al. (2015), Cameron and Buddle (2017)
16	*I. anglicana* → Other Araneae	0.15 ns		Agustí et al. (2003), Chapman et al. (2013)
17	OM → Eupodoidea	−0.53*		Liu et al. (2015), Liao et al. (2016)
18	*I. anglicana* → *C. holmgreni*	0.28*	44/0	Agustí et al. (2003), Chapman et al. (2013)
19	*I. anglicana* → *N. rufescens* larvae	−0.26*	14/0	Bilde, Axelsen, and Toft (2000), Eitzinger and Traugott (2011)
20	DS → *C. holmgreni*	−0.17 ns		Hein et al. (2014)
21	Chironomidae → *N. rufescens* larvae	0.18 ns	0/12.5	Hågvar and Ohlson (2013), Hågvar and Pedersen (2015)
22	Chironomidae → *C. holmgreni*	0.21*	56/0	Harwood, Sunderland, and Symondson (2003), Gratton, Donaldson, and Vander Zanden (2008), Hambäck et al. (2016), Sanchez‐Ruiz et al. (2018)
23	Other Araneae → *C. holmgreni*	0.22 ns	94/100	Cuff et al. (2021), Michalko et al. (2021)
24	Eupodoidea → Bdelloidae	0.31*		Novel
25	Eupodoidea → *N. rufescens* larvae	−0.47***	7/0	Novel
26	NDVI → *C. holmgreni*	0.55***		Diehl et al. (2013), (Gravesen 2008)
27	*I. anglicana* → *M. morio*	−0.06 ns	50/19	Hvam and Toft (2005), Hambäck et al. (2021)
28	*I. anglicana* → *N. rufescens* adults	−0.21 ns	25/12.5	Bilde, Axelsen, and Toft (2000)
29	Chironomidae → *N. rufescens* adults	0.38**	0/7	Hågvar and Ohlson (2013), Hågvar and Pedersen (2015)
30	Chironomidae → *M. morio*	0.28***	75/75	Harwood, Sunderland, and Symondson (2003), Gratton, Donaldson, and Vander Zanden (2008), Hågvar and Pedersen (2015)
31	*C. holmgreni* → *A. borealis*	0.79***		Coulson, Hodkinson, and Webb (2003b), Punzo and Ludwig (2005), Punzo (2006), Uma and Weiss (2010)
32	Other Araneae → *A. borealis*	−0.11 ns		Miliczky and Calkins (2001)
33	Other Araneae → *M. morio*	0.02 ns	25/6	Sergeeva (1999), Halaj and Cady (2000), Hambäck et al. (2021)
34	*C. holmgreni* → *M. morio*	0.35***	37.5/6	Sergeeva (1999), Halaj and Cady (2000), Hambäck et al. (2021)
35	*N. rufescens* larvae → *M. morio*	0.32***	56/87.5	Halaj and Cady (2000)
36	TSD → *M. morio*	0.36***		Novel
37	DS → *M. morio*	0.2 ns		Novel
38	Bdelloidae → *M. morio*	0.3***	0/12.5	Hambäck et al. (2021)
39	OM → *N. rufescens* adults	−0.45***		Gereben (1995)

Abbreviations: D.S., distance to the glacier snout; O.M., organic matter; S.W., soil water; T.S.D., time since deglaciation. Level of significance is indicated by: ns: not significant; *: *p* < 0.05; **: *p* < 0.01; ***: *p* < 0.001.

**TABLE A7:** Pathway statistics of SEM2. Pathways (Figure 9), standardized regression weights, significance levels, the % gut content represents the percentage of predators that have a specific prey species in their gut out of the total number of predator individuals examinedand literature references supporting the presumed process.

Number of pathway in SEM diagram	Pathway	Standardized regression weights and significance levels	Gut content % in vegetation/bare soil habitats	References
1	A–D of prey → IGP of *C. holmgreni*	−1.22***		Lucas and Rosenheim (2011)
2	A–D of prey → A–D of *N. rufescens*	−0.61***	27/15	Bilde, Axelsen, and Toft (2000), Eitzinger and Traugott (2011)
3	A–D of prey → A–D of *C. holmgreni*	0.52***	63/0	Harwood, Sunderland, and Symondson (2003)
4	A–D of *M. morio* → A–D of *N. rufescens*	−1.13***	27/45	
5	A–D of other Araneae → A–D of *C. holmgreni*	0.92***	94/100	Cuff et al. (2021)
6	A–D of other Araneae → A–D of *N. rufescens*	0.79***	20/10	Dawey et al. (2013)
7	A–D of *M. morio* → A–D of other Araneae	0.59**	50/12.5	

Abbreviations: A–D, activity–density; IGP, intra guild predation. The level of significance is indicated by: **: *p* < 0.01; ***: *p* < 0.00.1.

**TABLE A8:** Mean activity‐density arthropod numbers in wet pitfalls. Mean pitfall activity‐density numbers of arthropods over the seasons in the wet pitfall traps in vegetation and bare soil patches in the central area during the two study years as well as total numbers captured at the upper and lower transect in 2016.

Order	Suborder/family	Species	2015 Central	2016 Central	2016 Upper	2016 Lower
			*n* = 21	*n* = 14	*n* = 5	*n* = 4
Coleoptera	Carabidae	*Nebria rufescens*	14.9	18.4	11.2	16.0
		Adults	14.7	13.9	9.8	12.0
		Larvae	0.2	4.5	1.4	4.0
Opiliones	Phalangiidae	*Mitopus morio*	32.8	40.9	14.0	54.3
Araneae	Linyphiidae	*Collinsia holmgreni*	5.9	4.4	1.2	0.5
		*Oreoneta frigida*	2.9	1.1	0.8	0.3
		*Improphantes complicatus*	0.8	0.4	0.0	0.3
		*Bathyphantes eumenis*	0.6	0.3	0.0	0.0
		*Oreonetides vaginatus*	0.2	0.1	0.0	0.5
		*Islandiana princeps*	0.2	0.1	0.0	0.0
		*Walckenaeria karpinskii*	0.0	0.0	0.2	0.3
		*Walckenaeria clavicornis*	0.05	0.0	0.0	0.0
		*Agyneta nigripes*	0.1	0.0	0.0	0.0
		*Mecynargus borealis*	0.0	0.0	0.0	0.5
		*Erigone arctica*	0.0	0.1	0.0	0.0
		*Centromerus arcanus*	0.0	0.1	0.0	0.0
		*Agyneta jacksoni*	0.0	0.0	0.0	0.0
		*Mecynargus paetulus*	0.0	0.0	0.0	0.3
	Lycosidae	*Pardosa groenlandica*	1.1	0.1	0.0	1.8
		*Arctosa insignita*	0.1	0.0	0.2	2.3
		*Pardosa hyperborea*	0.1	0.0	0.0	0.0
	Thomisidae	*Xysticus durus*	0.0	0.0	0.0	1.0
	Hahniidae	*Hahnia glacialis*	0.05	0.0	0.0	0.3
	Philodromidae	*Thanatus arcticus*	0.0	0.0	0.0	0.3
Collembola	Isotomidae	*Isotoma anglicana*	75	18.8	94.8	6.75
Diptera	Chironomidae		17.5	5.7	6.0	4.5
	Sciaridae		10	2.7	3.2	3.0
	Anthomyiidae		9.1	16.8	27.2	14.8
	Mycetophilidae		1.8	5.0	1.0	5.3
	Mycetophilidae	*Mycomya* sp.	0.0	0.1	0.0	0.0
	Muscidae		0.8	0.7	3.0	1.3
	Simuliidae		0.6	0.4	0.6	0.5
	Culicidae		0.4	0.1	0.0	0.0
	Empididae		0.4	0.2	0.0	0.0
	Cecidomyiidae		0.4	0.1	0.0	0.0
	Tipulidae		0.1	0.4	0.2	0.0
	Phoridae		0.1	0.1	0.2	0.0
	Dolichopodidae		0.1	0.0	0.0	0.0
	Agromyzidae		0.1	0.0	0.0	0.0
	Scatophagidae		0.1	0.0	0.0	4.0
	Ceratopogonidae		0.05	0.0	0.2	0.0
	Calliphoridae		0.0	0.1	0.0	1.5
	Keroplatidae		0.0	0.3	0.0	0.0
	Trichoceridae		0.0	0.1	0.0	0.0
Trichoptera			0.0	0.1	0.0	0.0
Hemiptera	Aphididae	*Thecabius populimonilis*	4.9	2.0	0.0	3.3
Actinedida	Erythraeoidea		8.9	14.8	31.4	26.5
	Eupodoidea		8.1	0.0	0.4	0.0
	Bdellidae		5.7	3.9	13	3.3
	Tetranychidae	*Bryobia* sp.	4.1	1.1	0.9	0.5
Mesostigmata	Gamasida		0.0	0.6	0.8	1.5
Trichoptera			0.0	0.1	0.0	0.0
Hymenoptera	Ichneumonidae	*Aclastus borealis*	1.5	0.9	0.0	0.0
		*Barycnemis bellator*	0.1	0.0	0.0	0.0
		*Phygadeun* sp.	0.0	0.2	0.0	0.0
		*Orthocentrini* sp.	0.0	0.1	0.0	0.0
		*Plectiscidea sp*.	0.05	0.0	0.0	0.0
	Braconidae	*Microplitis coactus*	0.2	0.0	0.0	0.0
	Pteromalidae	*Ardilea convexa*	0.05	0.0	0.0	0.0
	Eulophidae	*Hemiptarsenus unguicellus*	0.05	0.0	0.0	0.0
		*Chrysocharis cf. pubicornis*	0.05	0.0	0.0	0.0
	Diapriidae	*Zygota ruficornis*	0.05	0.0	0.0	0.0

Abbreviation: *n*, number of traps.

**TABLE A9:** Kobbefjord spider catches. Linyphiid species caught in wet pitfall traps in Empetrum vegetation plot (1) and in Salix vegetation plot (3) in the summer of 2015 and the summer of 2016.

Date	Plot	Pitfall/sektion	*Pocadicnemis americana*	*Agyneta jacksoni*	*Ceratinella ornatula*	*Tiso aestions*	*Mecynargus morulus*	*Oreonetides vaginatus*	*Improphantes complicatus*
15‐07‐2015	1	D		♀					
15‐07‐2015	3	D	♀						
21‐07‐2015	1	B		♂					
28‐07‐2015	3	B		♀					
28‐07‐2015	3	B		♀					
28‐07‐2015	3	B		Subadult					
28‐07‐2015	3	C				♂			
28‐07‐2015	3	D		♂					
06‐08‐2015	3	D					♀		
19‐08‐2015	3	A	♂						
25‐08‐2015	1	D			♂				
25‐08‐2015	3	D	♀						
19‐05‐2016	3	B				♂			
19‐05‐2016	3	B				♂			
19‐05‐2016	3	B		♂					
19‐05‐2016	3	B		♂					
19‐05‐2016	3	B		♀					
19‐05‐2016	3	B	♂						
19‐05‐2016	3	B	♂						
19‐05‐2016	3	B	♂						
19‐05‐2016	3	B	♀						
19‐05‐2016	3	B	♀						
19‐05‐2016	3	B	♀						
19‐05‐2016	3	B	♀						
12‐07‐2016	3	D				♀			
22‐07‐2016	3	C	♂						
22‐07‐2016	3	D				♀			
27‐07‐2016	3	A	♀						
27‐07‐2016	3	A	Juvenile						
27‐07‐2016	3	C	♀						
02‐08‐2016	3	D	♂						
02‐08‐2016	3	D	Juvenile						
02‐08‐2016	3	D					♀		
23‐08‐2016	3	B						♀	
23‐08‐2016	3	D					♀		
01‐09‐2016	3	C	♂						
01‐09‐2016	3	C	♀						
01‐09‐2016	3	C							♀

**TABLE A10:** Ecological relations for SEM analysis, SEM3, of a wheat‐clover bi‐cropping experiment. Relations between web density, vegetation density, juvenile production, Collembola density, organic matter, and soil water in top‐soil. The experiment was conducted in an agricultural field in Denmark 1994–1995. For further explanations see (Gravesen 2008).

	Pathway	Standardized regression weights and significance levels	References
1	Web density → juvenile production	−0.23**	Van Baarlen, Sunderland, and Topping (1994), Walzer, Paulus, and Schausberger (2006), Walzer and Schausberger (2011, 2012), Montserrat et al. (2012)
2	Collembola density → Juvenile production	0.27**	Agustí et al. (2003), Marcussen, Axelsen, and Toft (2003)
3	Organic matter → Collembola density	0.36***	Lawrence and Wise (2000, 2003), Liu et al. (2015), Liao et al. (2016)
4	Soil water → Collembola density	0.31***	Liao et al. (2016)
5	Vegetation density → Organic matter	0.26**	Pérez (2001)
6	Collembola density → Web density	0.14 ns	Harwood, Sunderland, and Symondson (2003)
7	Vegetation density → Web density	0.13 ns	Gómez, Lohmiller, and Joern (2016)

### AM1. Geographic Location Information

Detailed Geographical Coordinates and Elevation:

- The transect at Qassinnguit mountain began at 206 m above sea level (MASL) and extended to 291 MASL, with specific coordinates at lat 64.1755, lon −51.3794.
- The study site on the northwestern slope of the Qassi mountain covered an elevation range of 250–330 MASL, with coordinates at lat 64.1575, lon −51.2913.
- The crowberry, *Empetrum nigrum* L., dominated site in Kobbefjord Valley was at 23 MASL with coordinates at lat 64.1339, lon −51.3839.
- The gray willow, *Salix glauca* L., dominated site was at 22 MASL with coordinates at lat 64.1316, lon −51.3714.

Data from Kobbefjord are available from Greenland Ecosystem Monitoring (2020b).

### AM2. Environmental Measurements

In the middle of each patch at the Qassinnguit glacier foreland, a soil sample was taken from the topsoil (5 cm in diameter and 3 cm in depth) for environmental characterization by measuring the percentage of actual soil water content and organic matter content during dry weather periods in both the summer of 2015 and 2016. Stones, roots, and leaves were removed by sifting the soil samples with a 2 mm sieve. Soil water content was analyzed by oven drying samples at 60°C for 24 h (Memmert oven model 600). After measuring the soil water content, the dry soil was analyzed for organic matter content at 600°C for 24 h.

The NDVI measurements were done using a Holland Scientific Crop Circle ACS‐210 Plant Canopy Reflectance Sensor (www.hollandscientific.com) (Raundrup et al. 2019). The Crop Circle sensor (Shaver et al. 2011) generates light with a wavelength of 590 nm in the VIS band (visible light wavelength) and 880 nm in the NIR band (near infrared wavelength). The amount of VIS and NIR light that is reflected by the plant canopy was measured and the NDVI value was calculated as follows: NDVI = (NIR − VIS)/(NIR + VIS). VIS is primarily dependent on the chlorophyll contained in the palisade layer of leaves and NIR reflectance depends on the structure of the mesophyll and the cavities between these cells. Therefore, greater leaf area and green plant biomass levels result in higher reflectance and higher subsequent NDVI values which means that dense vegetation has a high NDVI value while bare soil areas will have low NDVI values.

We estimated the time since permanent snow‐ or ice‐cover by lichenometric dating using *Rhizocarpon* thalli growth at the nearby Kangerlussuaq location, based on the time‐calibrated lichen growth curve of Forman et al. (2007). Twenty‐three lichen diameter measurements of *Rhizocarpon* lichen species colonies growing on stones and rocks were made and converted to age according to Forman et al. (2007), assuming linear growth above 20 mm diameter (Table A2; Figure A1). Based on this lichenometric dating, deglaciation of the upper part of the Qassinnguit transect was estimated to be between 1 and 260 years before present (BP), the central area was estimated to be between 45 and 2500 years BP, and the lower part was estimated to be between 820 and 1975 years BP (Figures A2, A4, A9).

### AM3. Arthropod Sampling

In the summer of 2015, a central transect of the Qassinnguit foreland (242–277 MASL) was surveyed. This was done using a combination of wet and dry pitfall traps across 21 patches. The wet traps consisted of 9 cm diameter, 14 cm deep plastic cups, which were half‐filled with a 20%–25% ethylene glycol solution to preserve captured arthropods. Detergent was added to break the surface tension of the solution.

The size of a patch was approximately 2 × 2 m separated from other patches by boulders. The 21 patches were classified according to their type of surface cover: pioneer vegetation, gravel or bare soil, see photos of the soil surface in Figure A1. Each cover type was investigated in seven patches each with a wet and a dry pitfall trap 1–2 m apart.

During the summer of 2016, patches with vegetation and bare soil cover were surveyed using the same pitfall trap setup as in 2015. A transect was extended above and below the central transect with wet pitfall traps. The upper (277–291 MASL) and lower (206–242 MASL) parts of the transect had five patches and four patches each, respectively, each with one wet pitfall trap (Figure 1). The upper transect patches had bare soil cover with just a little or no sunlight as they were in the shadow of the sun—even at midsummer (see photos in Figure A1). Two of the upper transect patches were covered by a mostly permanent snow layer before the summer of 2016 and were situated just next to the glacier snout consisting of permanent snow or ice, hereafter referred to as the glacier snout. The lowest transect patches had a climax vegetation cover, mainly with crowberry, which is a common heather plant (family Ericaceae) in Greenland.

During the summer of 2014, wet pitfall traps were used to survey a 259‐m long transect at a glacier foreland on a northwestern slope of the Qassi mountain (Figure 1). The 14 wet pitfall traps used at that transect were similar to the wet pitfall traps used at the Qassinnguit mountain during the summer of 2015 and the summer of 2016.

Two climax vegetation sites in the Kobbefjord Valley were surveyed during the same summer periods as at the glacier forelands of the Qassinnguit and the Qassi mountains from the summer of 2015 till the summer of 2017 (Greenland Ecosystem Monitoring 2020a). Each of these two climax vegetation sites were sampled using four pitfall traps that were half‐filled with salt water. Each of these pitfall traps were yellow plastic cups—10 cm in diameter and 8 cm deep. The climax vegetation sites in the Kobbefjord Valley were dominated by crowberry or gray willow. We believe that the differences in the design of the pitfall traps used in the glacier forelands (transparent and relatively deep) compared to those used in the Kobbefjord Valley (yellow and relatively shallow) do not pose a significant problem for comparing samples between the Qassinnguit foreland and the Kobbefjord Valley (Brown and Matthews 2016).

The wet pitfall traps were emptied at fortnightly intervals in the Qassinnguit and Qassi glacier forelands and at weekly intervals in Kobbefjord Valley during the sampling period, which started in early July and ended in mid‐August each year. All arthropods sampled in the wet pitfall traps were identified to species or family level.

The dry pitfall traps (cups of diameter 9 cm, 14 cm deep) were used to collect arthropods for molecular gut content analysis at the Qassinnguit glacier foreland during the summer of 2015 and the summer of 2016. These were emptied every 24 h to minimize the chance of contamination (Shokralla, Singer, and Hajibabaei 2010). They were half‐filled with cylindrical Styrofoam chips of length 2 cm and diameter 1 cm to aid prey escaping from predators in the traps similar to methodology by Raso et al. (2014) and Branco Leote, Rennstam Rubbmark, and Traugott (2024) using wood chips. Each live arthropod predator was put in a 1.5 mL micro centrifuge plastic tube filled with 95% ethanol. All samples were frozen at −20°C until subsequent DNA analysis. Between each sampling, all dry pitfall traps were washed with water and soap to avoid contamination from prior trapped arthropods, soil particles, etc. The dry pitfall traps had X‐shaped guidance barriers to improve the effect of the sampling (Boetzl et al. 2018).

**TABLE A11:** Sources of arthropod data collected for the study, including the years in which the data were collected, their specific locations, the corresponding successional stages of the habitats, the specific analyses conducted and replication per habitat. A. Wet pitfall traps. B. Dry pitfall traps.

A. Analyses of arthropod activity‐density data obtained by wet pitfall traps				
Year	Location	Successional stages/habitats	Analyses	Replication per habitat
2014	Qassi Glacier Foreland	Transect from bare soil over pioneer vegetation to climax vegetation	Nonlinear regression (Figure 4)	14
2015	Qassinnguit Glacier Foreland	Pioneer vegetation, gravel, and bare soil	Population comparison, SEM1	7
2015	Kobbefjord Valley^a^	Climax vegetation (crowberry and gray willow)	Population comparison, NDVI regression analysis	4
2016	Qassinnguit Glacier Foreland	Bare soil, vegetation, and climax vegetation	Nonlinear regression (Figures 2 and 4), SEM2	7
2016	Kobbefjord Valley^a^	Climax vegetation (crowberry and gray willow)	Population comparison, NDVI regression analysis	4
2017	Kobbefjord Valley^a^	Climax vegetation (crowberry and gray willow)	Population comparison	4

B. Metabarcoding gut content analysis of DNA from predators from dry pitfall traps. SEM: Structural Equation Modeling			
Year	Location	Successional Stage	Analyses
2015–2016	Qassinnguit Glacier Foreland	Pioneer Vegetation, Gravel, Bare Soil	SEM1 and 2, Trophic interaction analysis

^a^ Greenland Ecosystem Monitoring (2020a).

### AM4. Detailed Methodology for DNA Metabarcoding Gut Content Analysis and Bioinformatics for 2016

#### DNA Extraction and Analysis

##### DNA Extraction

DNA was extracted from predators and their potential prey using the DNeasy Blood and Tissue Kit (Qiagen), adhering to the standard protocol for animal tissue. Specific dissection methods isolated the gut or relevant digestive parts for adult ground beetles ( *N. rufescens* ) and harvestmen ( *M. morio* ), while whole‐body extractions were performed on beetle larvae. For spiders ( *C. holmgreni* ), extractions focused on the opisthosoma (Macías‐Hernández et al. 2018). The numbers of predators used for gut content analysis per species were as follows: *C. holmgreni* (17), *M. morio* (32), *N. rufescens* adults (32), and *N. rufescens* larvae (18). Cf. DRYAD repository for metabarcoding gut content results.

#### Metabarcoding Analysis

##### Sample Selection and Primer Testing

Ninety‐nine predators were selected for metabarcoding analysis using modified primers that amplify a broad range of prey. In silico testing confirmed effective amplification rates for most groups, except aphids. Sequencing was performed using Illumina NextSeq, and subsequent data processing involved merging reads, trimming, and clustering into operational taxonomic units (OTUs) with specific sequence divergence thresholds. Taxonomic assignments were based on sequence similarity to known references in the NCBI and BOLD databases.

#### Quality Control and Data Validation

##### Sequence Validation

Post‐sequencing processes removed erroneous sequences and rare haplotypes. Sequences were validated against known taxonomic data, with non‐native taxa to Greenland being excluded from the analysis (Böcher et al. 2015). This meticulous validation ensured the trophic interactions observed were accurate and representative of the local ecological context.

#### Blocking and PCR Specifics

##### Blocking Oligonucleotides

To reduce predator DNA amplification, blocking oligonucleotides with a C3‐spacer modification at the 3′ end were designed:

BlkNebria: 5′‐TWCCTGCTCCTCTTTCTACTATTGAACTT for *N. rufescens* and BlkMitopus: 5′‐TCCAGCCCCATTTTCTACTATTGTAGAA for *M. morio* . The specificity of these primers was tested using FastPCR (Kalendar et al. 2017). These oligos effectively reduced predator DNA amplification in laboratory testing, with potential blocking of one mite species (*Tetranychus* sp.) from the wide range of tested taxa, based on in silico tests.

##### PCR Cycling Conditions

Total DNA concentration was normalized to 10–20 ng/μL. A 158 bp long amplicon was amplified using MiteMinBar primers:

MiteMinBarF: 5′‐CATGCITTYRTIATRATTTTTTTYATAG, (de Groot, Laros, and Geisen 2016) and MiteMinBarRModif: 5′‐GGRTAAACWGTTCAWCCWGTWCC, which is a modified reverse primer designed particularly for the present study and then using a Multiplex PCR Kit (QIAGEN). The PCR cycling conditions were 15 min at 95°C followed by 40 cycles of 94°C for 30 s, 49°C for 90 s, 72°C for 90 s, with a final extension at 72°C for 10 min. PCR products were visualized on 2% agarose gels stained with ethidium bromide.

##### PCR Product Purification and Indexing

PCR products were purified using HighPrepTM PCR Clean Up (MAGBIO) magnetic beads at a ratio of 0.8 (21 μM of the PCR product and 16.8 μL of MagBio beads). Indexed PCR was performed with Nextera adaptors (Illumina), and different combinations of Nextera indexes in each sample allowed distinguishing prey from each individual predator. The reaction mixture consisted of 12 μL of AccuPrime SuperMix II, 2 μL of indexed primers (P5 and P7), 7 μL of ultra‐clean water, and 5 μL of the purified PCR product. The indexed PCR products were visualized on 2% agarose gels stained with ethidium bromide to check if the indexes attached to the amplicons. The PCR products were purified again with magnetic beads (0.8× volume), and the concentration of the purified PCR products was measured using Qubit.

### AM5. Barcoding of Species and Targeted Single‐Plex PCR for Family‐Level DNA Gut Content Analysis in Arthropod Predators

#### AM5.1. DNA Barcoding

DNA barcoding of the sampled arthropods was conducted to enhance the COI reference library used for the metabarcoding of predator gut contents. COI barcoding was performed at the Canadian Centre for DNA Barcoding (CCDB) for most of the specimens, as indicated by the GREARnnn‐nn process ID numbers listed in Table A4. The PCR procedure, described by Ivanova and Grainger (2009), utilized primers provided for each BOLD record on http://boldsystems.org. DNA extraction and PCR were carried out at the Department of Ecoscience, Aarhus University, using the Sigma‐Aldrich REDExtract‐N‐Amp kit according to the manufacturer's protocol with primers LCO1490 and HCO2198 (Folmer et al. 1994). The PCR program included an initial 94°C for 3 min, followed by five cycles of 94°C for 30 s, 45°C for 40 s, 72°C for 1 min, then 35 cycles of 94°C for 30 s, 51°C for 40 s, 72°C for 1 min, and a final extension at 72°C for 10 min. Sanger sequencing of the amplicons was performed by Macrogen Europe B.V using the Applied Biosystems 3730XL. The sequences were subsequently deposited on BOLD (www.boldsystems.org) and GenBank. Details such as arthropod species names, BOLD barcode index number (BIN), and GenBank accession codes are included in Table A4.

#### AM5.2. DNA Gut Content Analysis of Arthropod Predators Performed at University of Kentucky in 2015

##### Sampling Methods

In 2015, alongside wet pitfall traps, dry pitfall traps were utilized to collect predators for DNA gut content analysis. These traps did not contain fluid and were half‐filled with plastic pieces to prevent in‐trap predation. Plexiglass guides were placed on both sides of the trap to increase the capture of live arthropods. The traps were operational for 24 h, and all arthropods were collected alive, following methods described by Harper et al. (2005).

##### Sample Preparation

Live arthropod predators such as ground beetles ( *N. rufescens* ), harvestmen ( *M. morio* ), and several spider species from the family Linyphiidae were collected from dry pitfall traps. Each individual was placed in 1.5 mL tubes filled with 95% ethanol. These samples were then shipped to the University of Kentucky and stored at −25°C until DNA extraction. The Linyphiidae spiders were identified by spider taxonomist Jørgen Lissner at The Natural Historical Museum in Aarhus, Denmark.

Similar dissection methods as for the 2016 samples were applied to *Nebria rufescens* (isolating the gut), *Mitopus morio* (excluding legs and mouth parts through longitudinal dissection), and spiders (extracting from the opisthosoma) (Macías‐Hernández et al. 2018). The diet analysis targeted a spectrum of prey groups, with *I. anglicana* as the primary focus among Collembola.

##### DNA Extraction and PCR Methods

A total of 117 predators, comprising 39 each of *N. rufescens* , *M. morio* , and Linyphiidae, underwent DNA extraction at the University of Kentucky using the DNeasy Blood and Tissue Kit (Qiagen). Specific dissection methods were employed:

- *N. rufescens* : Gut isolation.
- *M. morio* : Longitudinal dissection excluding legs and mouth parts.
- Spiders: DNA extracted from the opisthosoma.

The extracted DNA, including the contents of their guts, was screened for prey DNA by single‐plex PCR using order‐ and family‐level specific primers developed for earlier projects but specific to Collembola, Diptera, and Aphididae. The 2015 PCR protocols for these primers were as follows (primer source publications included):

- Collembola primers (COL4F and COL5R): 45 cycles at 94°C for 1 min, 61°C for 45 s, and 72°C for 45 s (Kuusk and Agustí 2008).
- Aphid primers (S421_aphids2F and A424_aphids1 + 2R): 45 cycles at 94°C for 1 min, 65°C for 45 s, and 72°C for 45 s (Staudacher, Jonsson, and Traugott 2016).
- Diptera primers (DIPS16 and DIPA17): 45 cycles at 94°C for 1 min, 60°C for 45 s, and 72°C for 45 s (Eitzinger et al. 2013).

PCRs included positive and negative controls and were conducted using Takara Ex Taq in Bio‐Rad thermal cyclers. The amplified DNA was visualized on 2% SeaKem agarose gels pre‐stained with GelRed nucleic acid gel stain.

##### Results

The DNA gut content analysis revealed that both ground beetles, *N. rufescens* , and harvestmen, *M. morio* , were multi‐channel feeders, consuming both detritivores (Collembola and Diptera) and a herbivore (Aphididae) (Figure A11). Collembola were commonly consumed by spiders and their consumption increased with their abundance in each habitat type. Ground beetles primarily consumed Collembola in gravel patches, whereas collembolan DNA was only detected in harvestmen from vegetated areas. Flies (Diptera) were consumed by all three predator types, with ground beetles consuming them most frequently. Curiously, aphid DNA was only detected in beetles and harvestmen from bare ground patches, where aphid abundance was very low and no vascular plants were present, suggesting possible aphid movement into these areas.
